# ACRIN 6684: Multicenter, phase II assessment of tumor hypoxia in newly diagnosed glioblastoma using magnetic resonance spectroscopy

**DOI:** 10.1371/journal.pone.0198548

**Published:** 2018-06-14

**Authors:** Eva-Maria Ratai, Zheng Zhang, James Fink, Mark Muzi, Lucy Hanna, Erin Greco, Todd Richards, Daniel Kim, Ovidiu C. Andronesi, Akiva Mintz, Lale Kostakoglu, Melissa Prah, Benjamin Ellingson, Kathleen Schmainda, Gregory Sorensen, Daniel Barboriak, David Mankoff, Elizabeth R. Gerstner, on behalf of the ACRIN 6684 trial group

**Affiliations:** 1 Department of Radiology, Neuroradiology Division, Massachusetts General Hospital, and Harvard Medical School, Boston, MA, United States; 2 A. A. Martinos Center for Biomedical Imaging, Charlestown, MA, United States of America; 3 Center for Statistical Sciences, Brown University, Providence, RI, United States of America; 4 Department of Radiology, University of Washington, Seattle, WA, United States of America; 5 Department of Radiology, Wake Forest University, Winston-Salem, NC, United States of America; 6 Department of Radiology, Mt. Sinai Medical Center, New York, NY, United States of America; 7 Department of Radiology, Medical College of Wisconsin, Milwaukee, WI, United States of America; 8 Department of Radiology, UCLA Medical Center, Los Angeles, CA, United States of America; 9 Department of Radiology, Duke University, Durham, NC, United States of America; 10 Department of Radiology, University of Pennsylvania, Philadelphia, PA, United States of America; 11 Massachusetts General Hospital Cancer Center, Boston, and Harvard Medical School, MA, United States of America; Instituto de Investigacion Sanitaria INCLIVA, SPAIN

## Abstract

A multi-center imaging trial by the American College of Radiology Imaging Network (ACRIN) “A Multicenter, phase II assessment of tumor hypoxia in glioblastoma using ^18^F Fluoromisonidazole (FMISO) with PET and MRI (ACRIN 6684)”, was conducted to assess hypoxia in patients with glioblastoma (GBM). The aims of this study were to support the role of proton magnetic resonance spectroscopic imaging (^1^H MRSI) as a prognostic marker for brain tumor patients in multi-center clinical trials. Seventeen participants from four sites had analyzable 3D MRSI datasets acquired on Philips, GE or Siemens scanners at either 1.5T or 3T. MRSI data were analyzed using LCModel to quantify metabolites N-acetylaspartate (NAA), creatine (Cr), choline (Cho), and lactate (Lac). Receiver operating characteristic curves for NAA/Cho, Cho/Cr, lactate/Cr, and lactate/NAA were constructed for overall survival at 1-year (OS-1) and 6-month progression free survival (PFS-6). The OS-1 for the 17 evaluable patients was 59% (10/17). Receiver operating characteristic analyses found the NAA/Cho in tumor (AUC = 0.83, 95% CI: 0.61 to 1.00) and in peritumoral regions (AUC = 0.95, 95% CI 0.85 to 1.00) were predictive for survival at 1 year. PFS-6 was 65% (11/17). Neither NAA/Cho nor Cho/Cr was effective in predicting 6-month progression free survival. Lac/Cr in tumor was a significant negative predictor of PFS-6, indicating that higher lactate/Cr levels are associated with poorer outcome. (AUC = 0.79, 95% CI: 0.54 to 1.00). In conclusion, despite the small sample size in the setting of a multi-center trial comprising different vendors, field strengths, and varying levels of expertise at data acquisition, MRS markers NAA/Cho, Lac/Cr and Lac/NAA predicted overall survival at 1 year and 6-month progression free survival. This study validates that MRSI may be useful in evaluating the prognosis in glioblastoma and should be considered for incorporating into multi-center clinical trials.

## Introduction

Glioblastoma (GBM) is the most common and, unfortunately, the most aggressive type of primary malignant brain tumor. Despite treatments with surgery, radiation, and chemotherapy, the median overall survival is less than 15 months [1]. One of the pathologic hallmarks of GBM is tumor hypoxia resulting from an inefficient blood supply [2]. Tumor hypoxia limits the efficacy of radiation and chemotherapy and may select for a more aggressive tumor phenotype. It may also be a potent stimulator of abnormal angiogenesis [3]. Thus, a multi-center imaging trial, “American College of Radiology Imaging Network (ACRIN) 6684: A Multicenter, phase II assessment of tumor hypoxia in Glioblastoma using 18F Fluoromisonidazole (FMISO) with PET and MRI”, was conducted to assess hypoxia in patients with GBM.

The study found that increased tumor hypoxia as measured by ^18^F-FMISO standardized uptake values (SUV) peak and increased tumor vascular permeability (as measured by dynamic contrast enhanced MRI) were significantly associated with shorter overall survival at 1 year (OS-1). Furthermore, increased tumor perfusion (as measured by dynamic susceptibility contrast MRI) and increased vascular permeability (k^trans^) were significantly associated with shorter progression free survival [4]. These findings highlight that hypoxia and abnormal tumor vasculature can have a significant impact on patient outcomes. Here, we report the results of the analysis of the proton magnetic resonance spectroscopic imaging (^1^H-MRSI) data for this trial in a subset of patients.

^1^H-MRSI is a magnetic resonance-based imaging modality that allows non-invasive sampling of metabolic changes in normal and abnormal brain parenchyma. It has also been proven useful in the diagnosis, prognosis, and management of brain tumor patients [5–12]. The most common metabolites evaluated in routine clinical practice include N-acetylaspartate (NAA), choline-containing compounds (Cho), lactate (Lac) and creatine (Cr).

N-Acetylaspartate is considered a neuronal metabolite and is decreased in processes with neuronal destruction or dysfunction [13]. NAA resonance is reduced or completely absent in malignant brain tumors as neurons are replaced by neoplastic tissue [14–16]. Choline is related to membrane turnover and their elevation is indicative of a process that results in increased glial proliferation and membrane synthesis (as seen with cellular proliferative disorders) [14, 17, 18]. Furthermore, an increase in Cho is directly related to tumor malignancy [19]. The dual effects of decreased NAA due to neuronal loss or dysfunction and increased Cho due to alterations in tumor membrane turnover make the NAA/Cho ratio a sensitive marker for brain tumors. Lactate is implicated in many cancer mechanisms: it is produced by enhanced glycolysis and glutaminolysis, it promotes cancer cell survival and proliferation, and it may also stimulate angiogenesis [20]. Consequently, the presence of Lac may indicate high level of malignancy [21, 22]. Creatine is a metabolite related to the cellular energy metabolism. It is considered relatively stable in different pathological processes affecting the central nervous system and useful as a reference metabolite. However, Cr is typically decreased in astrocytomas [16].

In this sub-study of the primary 6684 study, we sought to test whether the MRSI measures—e.g. NAA/Cho, Cho/Cr, Lac/Cr and Lac/NAA were predictive of outcome, and to evaluate how these MRSI measures relate to the previously analyzed MRI and PET markers.

## Materials and methods

### Study subjects

ACRIN, a cooperative group funded by the National Cancer Institute, conducted a prospective multi-institutional study in patients with newly diagnosed GBM (NCT00902577). The ACRIN 6684 trial enrolled 50 participants from 11 academic centers in the US between 2010 and 2013. Each participating site obtained approval by their local Institutional Review Board before subject accrual and obtained written informed consents for all subjects. Participants were referred by their treating neuro-oncologists or medical oncologists, who were made aware of the study by the site principal investigator, and were approached and consented by a member of the study team. All procedures performed in studies involving human participants were in accordance with the ethical standards of the institutional and/or national research committee and with the 1964 Helsinki declaration and its later amendments or comparable ethical standards. Forty-two patients had evaluable PET and MR imaging studies, and the results have been reported in the primary paper. [4].

Eligibility criteria included patients with a histological confirmed diagnosis of GBM with imaging verified residual disease after surgery who were scheduled to receive standard fractionated radiation therapy in addition to temozolomide chemotherapy. In addition to radiotherapy, patients could receive an investigational agent as part of a clinical trial. Other inclusion criteria included Karnofsky Performance Score > 60 and age > 18. Patients were followed every 3 months for tumor progression and survival for at least 1 year. The detailed inclusion and exclusion criteria can be viewed at *https://www.acrin.org/Portals/0/Protocols/6684/ACRIN6684_Amend7_012412_master_ForOnline.pdf* (section 6).

**Fig 1:**
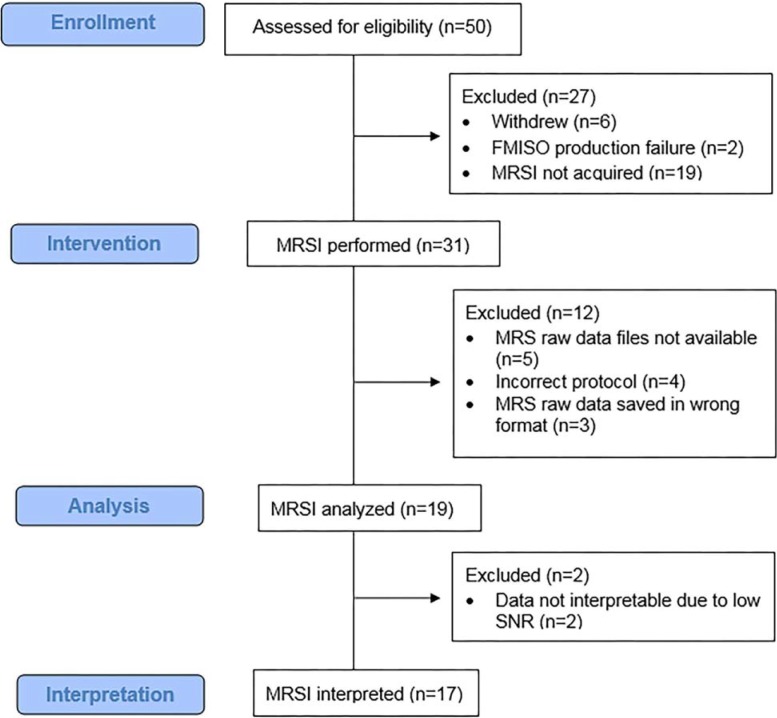
Consort flowchart. The ACRIN 6684 trial enrolled 50 participants, 27 participants were excluded due to withdrawal (n = 6), technical difficulty in ^18^F-FMISO production (n = 2) and because MRSI was not performed (n = 19). MRSI was attempted in 31 cases. 17 (55%) had evaluable MRSI data. Reasons for excluding data included: 1. MRS raw data had not been saved at the time of data acquisition (N = 5), 2. Incorrect protocol had been chosen (N = 4), 3. MRS raw data had not been saved in the right format (N = 3), and 4. Data were not interpretable due to low SNR (N = 2).

A subset of eight institutions initially qualified. MRSI was attempted in 31 cases. Of these 31 cases, 17 (55%) from four institutions had evaluable MRSI data. The 4 institutions, using 4 different MR scanners, collected MRSI data from the following numbers of patients: University of Washington (Philips 3T, 10 patients), Wake Forrest (GE 3T, 2 patients), Lee Moffitt (Siemens 1.5T, 4 patients), and Duke (Siemens 3T, 1 patient). The most common reasons for excluding data included: 1. The MRSI raw data files had not been saved at the time of data acquisition (N = 5), 2. The MRSI raw data had not been saved in the right format (N = 3) 3. Incorrect protocol has been chosen (N = 4), and 4. The data was not interpretable due to low SNR (N = 2) (Fig 1).

### MRI/MRSI acquisition

Research MRI and ^18^F-FMISO PET scans were performed after tumor diagnosis and within 2 weeks prior to initiation of chemo-radiation therapy. All sites were required to submit test scans to ensure image quality and the ability to perform required standardized image acquisition protocols prior to site participation The MRI requirements included anatomic pre-/post-contrast sequences along with dynamic contrast-enhanced (DCE) imaging, dynamic susceptibility contrast (DSC) perfusion, DTI, and MRSI. Acquisition and analysis of the DCE, DSC, and DTI data have been previously described [4]. The detailed MRI and PET imaging protocol can be viewed online at *http://www.acrin.org/Portals/0/Protocols/6684/ACRIN6684_Amend7_012412_master_ForOnline.pdf* (section 10).

Sites interested in participating in the exploratory study objectives related to MRSI were required to submit MRSI test data on phantoms and healthy subjects to the ACRIN core lab for quality control. All data were acquired using a 3D MRSI sequence with point-resolved spectroscopy excitation pulse sequence for signal localization. Acquisition parameters included TE = 140–144ms, TR = 1140–1180ms, phase encoding = 12x12x8, a FOV of >160 and nominal voxel sizes 2mL-3.5mL. Spectral bandwidth ranged from 1200 to 2000 Hz and number of points ranged from 512 to 1024 points. On the Philips scanner, 3 slices were acquired with 2D phase encoding of 12x11. The imaging time for MRSI was less than 10 minutes at all sites.

### MRSI data analysis

**Fig 2:**
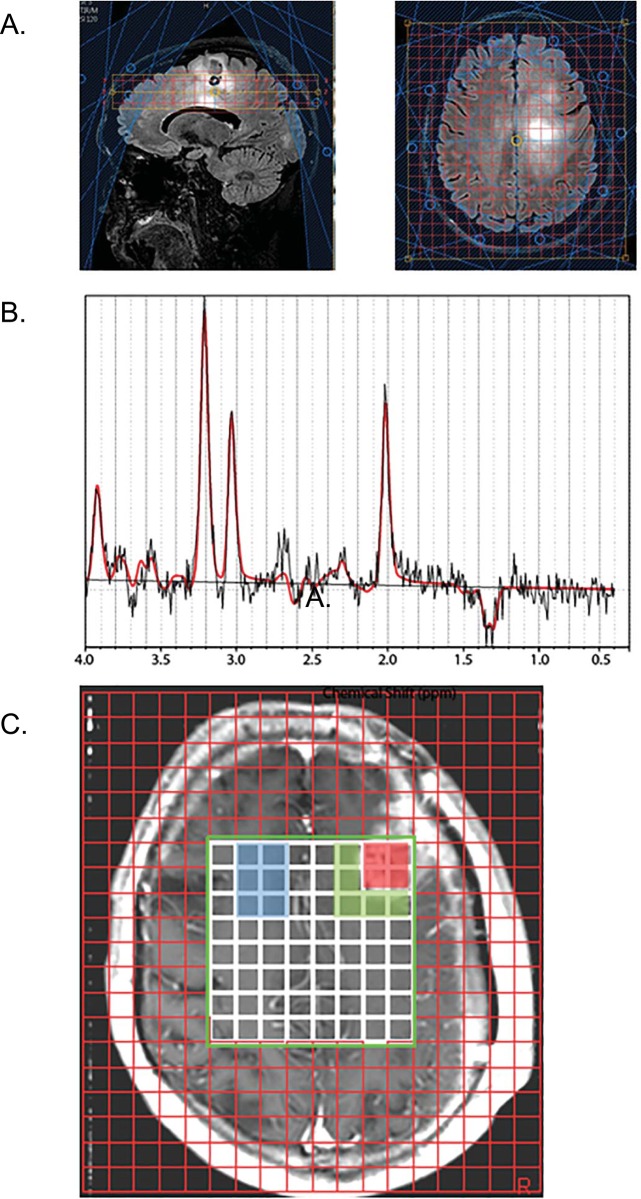
MRSI methods. *A*: Sagittal (left) and axial (right) MRSI voxel placement on a Philips scanner. Three slices were acquired with 2D phase encoding of 12x11. Data were acquired using a point-resolved spectroscopy excitation pulse sequence for signal localization. Acquisition parameters for all MRSI data included TE = 135–144 ms and TR = 1140–1180 ms. Also shown are the saturation bands for lipid suppression. *B*: The spectroscopic raw data were analyzed using LCModel 6.1 Software to determine the quantities of the metabolites NAA at 2 ppm, Cr at 3 ppm, Cho at 3.2 ppm, and Lac, a doublet, at 1.3 ppm. The gray line shows the spectrum; the red line indicates the fit of the MRS data. *C*: For spectra classification, MRSI data were overlaid on the post-contrast T1-weighted images. Voxels were classified into (i) enhancing tumor (red), (ii) non-enhancing peritumoral parenchyma approximately 1–1.5cm beyond the enhancing margin (green), and (iii) contralateral normal white matter (blue).

The spectroscopic raw data were centrally analyzed by ER who was blinded to the outcome of the participants. The data were analyzed using LCModel 6.1 Software [23] to determine the quantities of the metabolites NAA, Cr, Cho, and Lac. In addition, the presence of lipids (0.5–2 ppm) was recorded. Only fitted spectra with estimated standard deviations (Cramer–Rao lower bounds, automatically provided by the LCModel software) expressed in percent of the estimated concentrations lower than 25% were accepted [23]. In addition to the Cramer-Rao bounds criteria, spectra have been inspected by a spectroscopist for quality assurance. Data sets with low SNR (SNR <3) even on the contra lateral site were entirely excluded (N = 2). Also voxels containing subcutaneous fat from the scalp were excluded. Furthermore, MRSI datasets were vetted to ensure combability between the datasets as much as it was possible for a multi-center trial utilizing multiple vendors and magnetic field strengths. E.g. MRS datasets were excluded if they had been acquired using parameters that were outside the range of the stated protocol, e.g. 4 datasets were excluded because an incorrect echo time of TE = 45ms had been chosen. (Please see Results). For spectra classification, the MRSI data were overlaid on the post-contrast T1-weighted images. Voxels were classified into (i) contrast enhancing tumor; (ii) non-enhancing peritumoral parenchyma (periphery), and (iii) contralateral normal white matter, and mean metabolite ratios in those regions were calculated as previously described [5] (Fig 2).

### Statistical data analysis

Receiver operating characteristic (ROC) curves for the spectroscopic markers NAA/Cho, Cho/Cr, Lac/Cr, and Lac/NAA were constructed for two binary outcomes: OS-1 and 6-month progression free survival (PFS-6) as outlined under the study objectives/specific aims of the ACRIN 6684 trial (*https://www.acrin.org/Portals/0/Protocols/6684/ACRIN6684_Amend7_012412_master_ForOnline.pdf*). The areas under the ROC curves (AUC) and the associated 95% CIs were estimated empirically. A marker was considered effective in classification of an outcome status when its lower 95% CI of the AUC was at least 0.50. Correlations between parameters were assessed using Pearson correlation coefficients. All analyses were done with SAS 9.4 (Cary, NC, USA). Of note, the MRSI exam was a secondary and an exploratory aim in the study. Therefore, the study was not originally powered for this secondary objective. The data generated from this study may help us to accurately power future studies of MRS in glioblastoma.

## Results

### Study cohort

Patient demographic information, tumor size, O^6^-methylguanin-DNA-methyltransferase (MGMT) status, and initial treatment as well as salvage therapy received for the 17 evaluable patients are presented in Table 1. Of note, the patient characteristics in the 17 evaluable patients were similar to the entire cohort of 42 patients included in the primary paper [4]; e.g. the median age in this cohort was 59 y/o, the same as in the previous publication; the male gender percentage was 65% vs. 64%, and the median residual tumor size was 11.97 vs. 13.27. Furthermore, the OS-1 for the 17 evaluable patients was 59% vs. 60% in the larger cohort. These results suggest that the rejection or non-availability of some of the MRSI data has not introduced any significant bias into patient selection that might impact the analysis and results.

**Table 1:** Patient cohort.

	Total Evaluable N = 17
***Patient Characteristics***	
Median age (range)	59 (45,77)
Gender, male (%)	11 (65%)
Median residual CE tumor volume (range)	11.97 (0.84, 59.5)
MGMT methylated*	1 (6%)
MGMT unmethylated	3 (18%)
MGMT unknown/not tested	13 (76%)
***Initial Treatment***	
Temozolomide + RT	12 (71%)
Temozolomide + RT+ clinical trial drug	4 (23%)
Temozolomide +BSI (PARP inhibitor), but no RT	1 (6%)
***Salvage Therapy***^#^	
None	4 (24%)
RT	1 (6%)
Surgery	1 (6%)
Bevacizumab	4 (24%)
Chemotherapy	9 (53%)
NovoTTF	9 (53%)
***Survival***	
Median survival time, days (95%CI)	403 (209, 642)
OS-1 (%)	10 (59%)
Median PFS, days (range)	201 (128,335)
PFS-6 (%)	11 (65%)

CE contrast enhancement; RT radiation therapy

*Central review of tissue was not required and not all sites tested MGMT or patient underwent biopsy so there was insufficient tissue for MGMT testing.

# Patients could be counted more than once if received multiple salvage therapies.

### MRSI metabolic ratios as predictor of 1-year overall survival (OS-1)

**Fig 3:**
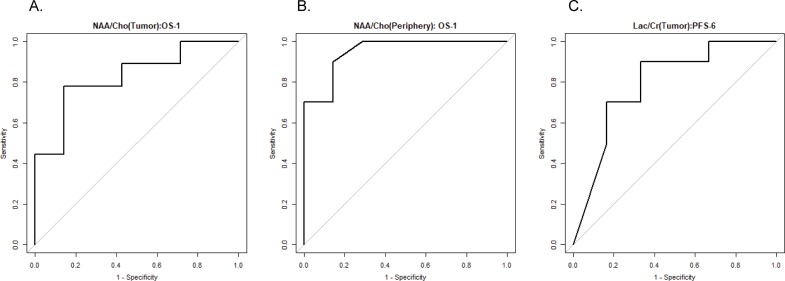
ROC curves with the corresponding AUC for predictors (A. tumor NAA/Cho, B. peripheral NAA/Cho, and C. tumor Lac/Cr) using 1-year overall survival (OS-1) and of 6-month progression free survival (PFS-6).

The OS-1 for the 17 evaluable patients was 59% (10/17). ROC analyses found the NAA/Cho in tumor (AUC = 0.83, 95% CI: 0.61 to 1.00) and in peritumoral parenchyma (AUC = 0.95, 95% CI 0.85 to 1.00) were predictive of survival at 1 year with higher values predicting increased OS-1 (Table 2). In addition, Lac/Cr in tumor showed a trend towards association with OS-1, with higher Lac values predicting poorer outcome (AUC = 0.78, 95% CI: 0.49 to 1.00). Cho/Cr was not effective in predicting OS-1. ROC curves with the corresponding AUC for the significant predictors using OS-1 are shown in Fig 3.

**Table 2:** Receiver operating characteristic (ROC) analysis.

Metabolic ratio	ROI	OS-1 AUC (95% CI)	PFS-6 AUC (95% CI)
NAA/Cho(n = 16)	Tumor	**0.83 (0.61–1.00)**	0.67 (0.39–0.95)
Cho/Cr(n = 16)	Tumor	0.65 (0.35–0.96)	0.60 (0.27–0.93)
Lac/Cr (n = 16)	Tumor	0.73 (0.45–1.00)	**0.79 (0.54–1.00)**
Lac/NAA(n = 16)	Tumor	*0*.*78 (0*.*49–1*.*00)*	*0*.*76 (0*.*49–1*.*00)*
NAA/Cho(n = 17)	Periphery	**0.95 (0.85–1.00)**	0.67 (0.40–0.95)
Cho/Cr(n = 17)	Periphery	0.63 (0.34–0.92)	0.53 (0.23–0.83)
Lac/Cr(n = 17)	Periphery	0.56 (0.25–0.86)	0.53 (0.26–0.80)
Lac/NAA(n = 17)	Periphery	0.57 (0.26–0.88)	0.53 (0.24–0.82)

ROI Region of Interest; OS-1 one-year overall survival; PFS-6 Six-month progression free survival; AUC area under the ROC curve; NAA N-acetylaspartate; Cr creatine; Cho choline-containing compounds; Lac lactate

Bold AUCs indicate significant predictions of outcome and *italics AUCs* indicate trends towards predicting outcome.

### MRSI metabolic ratios as predictor of 6-month progression free survival (PFS-6)

Six-month progression free survival was 65% (11/17). Neither NAA/Cho nor Cho/Cr was effective in predicting PFS-6 (Table 2). Lac/Cr in tumor was a significant negative predictor of PFS-6, indicating that higher Lac/Cr levels are associated with poorer outcome (AUC = 0.79, 95% CI: 0.54 to 1.00). In addition, Lac/NAA in tumor showed a trend as a negative predictor of PFS-6, indicating that higher Lac/NAA levels are associated with poorer outcome (AUC = 0.76, 95% CI: 0.49 to 1.00). ROC curves with the corresponding AUC for the significant predictors using PFS-6 are shown in Fig 3.

### Correlations between MRSI and other imaging markers

In addition, we explored the correlations between MRS markers and MR imaging markers of vascularity (CBV, CBF, and k^trans^) as well as between MRS markers and PET markers of tumor hypoxia (SUVmax). None of these markers were significantly associated with MRS markers (P >0.1) and Pearson correlation coefficient absolute values were below 0.42 (Table 3).

**Table 3:** Person Correlations between MRS markers and MRI markers of vascularity, CBV, CBF, and k^trans^) as well as between MRS markers and PET markers of tumor hypoxia (SUVmax).

Metabolic ratio	ROI	nrCBV (R)	nCBF (R)	Median k^trans^ (R)	SUV max (R)
**NAA/Cho**	Tumor	-0.38	-0.41	-0.08	-0.33
**Cho/Cr**	Tumor	0.24	0.28	-0.02	0.03
**Lac/Cr**	Tumor	-0.27	-0.25	-0.06	-0.29
**Lac/NAA**	Tumor	-0.02	-0.01	0.23	-0.18
**NAA/Cho**	Periphery	-0.33	-0.34	0.14	-0.41
**Cho/Cr**	Periphery	0.17	0.20	-0.27	0.11
**Lac/Cr**	Periphery	-0.09	-0.11	0.10	-0.15
**Lac/NAA**	Periphery	-0.01	-0.01	0.33	-0.04

(**nRCBV:** relative cerebral blood volume, corrected for leakage effects and normalized to normal-appearing white matter; **nCBF:** cerebral blood flow, normalized to normal-appearing white matter; **k**^**trans**^: vascular permeability; SUV standardized uptake value

## Discussion

This study reports the results of the ^1^H-MRSI data of the ACRIN 6684 multi-center imaging trial to assess tumor hypoxia in GBM using ^18^F-FMISO PET and MRI. We previously showed that increased tumor hypoxia as measured by ^18^F-FMISO SUVpeak was significantly associated with shorter OS-1. Furthermore, increased tumor perfusion (rCBV and rCBF,) and increased vascular permeability (median k^trans^) were significantly associated with shorter PFS [4]. Since MRSI is able to measure metabolites that reflect tumor burden, it may be able to provide additional information to the previously reported MRI and PET parameters.

Our data suggest that the MRS marker NAA/Cho in the tumor and in the peritumoral periphery were significant predictors of survival. NAA/Cho or its inverse Cho/NAA have previously been described as reliable biomarkers of tumor prognosis and biomarkers for treatment response [7–10]. In our study, peritumoral NAA/Cho showed the highest prediction of OS-1. This may indicate that increased tumor infiltration into the peritumoral parenchyma reflects active tumor growth and subsequently poorer outcomes [24]. On the other hand, tumor spectra within the contrast enhancement reflect less active tumor where growth is stalled because of necrosis, lack of blood supply and/or hypoxia. These results also emphasize that MRSI, compared to a single voxel approach, is essential in the evaluation of brain tumors as it simultaneously covers multiple areas of the tumor environment.

In our study, increase in Lac/Cr in the tumor was a marker of poor prognosis for PFS-6 and increases in Lac/NAA in the tumor showed a trend associated with poor prognosis for PFS-6 and OS-1. The production of lactate, tumor acidosis, and hypoxia are commonly thought to be linked. However, some studies have shown that high lactate concentrations are not necessarily associated with hypoxia [20, 25, 26]. Cancer cells will produce Lac in the absence of hypoxia. Low blood perfusion rates, aerobic glycolysis, and increased glutaminolysis may also contribute to lactate accumulation in non-hypoxic areas [20, 27–29]. With the entire cohort of 42 patients included in the primary paper [4], our group showed that increased tumor hypoxia as measured by ^18^F-FMISO SUVpeak was significantly associated with poor outcome. This study, on a subset of the same subjects, shows increased Lac/Cr levels were significantly associated with shorter PFS-6. Interestingly, no correlation between Lac/Cr and ^18^F-FMISO SUVpeak was observed in this study, which could be due to a limited sample size, measured values assessed in different locations within the tumor and peritumoral parenchyma, or the possibility that both markers provide complementary information.

Thus, the lack of correlations between metabolic markers measured by MRSI, hypoxia measured by ^18^F-FMISO PET, and MRI markers of tumor perfusion (e.g. nrCBV, nrCBF and median K trans) remains an interesting area for further study. The interactions between perfusion, hypoxia, and metabolism reflect the complexity of tumor biology and likelihood of response to chemotherapy or radiation. The lack of correlation may also be attributable to the limited sample size of 17 patients. Currently, we have too few data to study whether the combination of hypoxia information from ^18^F-FMISO PET and lactate information from MRSI might be complementary and helpful. Thus, complementary multimodal PET and MR imaging that captures diverse biological information simultaneously may be needed to tease out the complexities of tumor biology and may warrant further investigations.

One of the major limitations of this study is the limited sample size, with only 17 out of 31 cases having evaluable MRSI data. However, the rejection of some of the MRSI data does not appear to have introduced significant bias into the analysis since this smaller subset was similar to the larger cohort based on demographic and survival characteristics. Another limitation of this study pertains to possible partial volume effects because each voxel may contain partial volume fractions of more than one tissue type. Finally, the MRSI scans in our study came from 4 different institutions with different magnetic field strengths and with 3 different vendor magnets and MRSI acquisition software packages, which may limit comparisons.

Clinical multi center trials that use MRS as a tumor biomarker have proven to be challenging [30]. Typical challenges include the spectral quality of MRS data including low SNR, incorrect voxel placements at the time of scan acquisition, volume averaging due to large voxel sizes (averaging of surrounding benign tissue), and insufficient separation of metabolite peaks, as well as inadequate water suppression due to suboptimal shimming [5, 18]. Specifically, from our study sites, the most common reasons for excluding data were the logistical difficulties where MRSI raw data files had not been saved or sites had not adhered to the acquisition protocol. Only 2 datasets were not interpretable due to low SNR. Consequently, better education and closer monitoring during clinical trials are needed to increase the number of analyzable MRSI data sets. For example, site visits by a MR spectroscopist or a point person at a site may help educating MR technologists to improve MRSI data acquisition and saving of the datasets. Finally, the implementation of state-of-the art MRSI methods that run with minimal user interaction may also help to alleviate these challenges which calls for more effort from the MRS user community and vendors to implement these advanced tools in clinical protocols.

However, despite these challenges, there have been several successful clinical trials using MRS as biomarkers [31]. One of the utilizations of MRSI in multi-center clinical trials pertains to the automatic classifications of brain tumors from MR spectra in adults [21, 32–37] and pediatric populations [38, 39]. Furthermore, MRSI has been used for prognoses in patients with glial tumors [40] or for therapeutic monitoring to determine the efficacy of medications [5]. In a prior trial by ACRIN (Protocol RTOG 0625/ACRIN 6677 “A Randomized Trial of Bevacizumab with Temozlomide in Recurrent Glioblastoma”), our group reported that increased levels of NAA/Cho and decreased levels of Cho/Cr at 8 weeks post bevacizumab-based therapy in patients with recurrent GBM correlated with PFS-6 and OS-1 [5].

In summary, despite the small sample size in the setting of a multi-center trial comprising different vendors, field strengths, and varying levels of expertise at data acquisition, MRS markers NAA/Cho, Lac/Cr and Lac/NAA predicted OS-1 and PFS-6 well. Thus, given the positive results from both ACRIN6677 and ACRIN 6684, optimizing MRSI acquisition for implementation of these promising biomarkers appears warranted in evaluating patients’ prognosis. More sophisticated analysis approaches using machine learning techniques to look at the entire spectra rather than individual metabolites from MRS and MRSI also hold great promise to build on our promising results.

## Conclusion

Despite all aforementioned challenges, ^1^H MRSI measures were predictive of outcome in a group of patients with GBM treated using radiotherapy and temozolomide with information that appears distinct from and complementary to DCE/DSC-MRI and hypoxia PET. Therefore, standardized MRSI should be considered for incorporation into future multi-center clinical trials and may be useful in diagnosis and in risk stratifying patients.

## Supporting information

S1 Table Transparent reporting of evaluations with nonrandomized designs (TREND) checklist. (PDF)

S1 File Trial Study protocol for the American College of Radiology Imaging Network (ACRIN). “Multicenter, phase II assessment of tumor hypoxia in glioblastoma using ^18^F Fluoromisonidazole (FMISO) with PET and MRI”.
(PDF)
