# Supplementary material for: ACRIN 6684: Multicenter, phase II assessment of tumor hypoxia in newly diagnosed glioblastoma using magnetic resonance spectroscopy
Source: PLoS One. 2018 Jun 14;13(6):e0198548. doi: 10.1371/journal.pone.0198548 (PMC6002091; doi:10.1371/journal.pone.0198548)
Supplement: S1 File — “Multicenter, phase II assessment of tumor hypoxia in glioblastoma using 18F Fluoromisonidazole (FMISO) with PET and MRI”. (PDF) [file pone.0198548.s002.pdf]

AMERICAN COLLEGE OF RADIOLOGY IMAGING NETWORK

ACRIN 6684

MULTICENTER, PHASE II ASSESSMENT OF TUMOR HYPOXIA IN GLIOBLASTOMA USING ^{18}F-FLUOROMISONIDAZOLE (FMISO) WITH PET AND MRI

Investigational Agent Name: Fluorine-18 Fluoromisonidazole (^{18}F-FMISO)
Agent NSC Number: NSC 742836
Cancer Imaging Program, DCTD, NCI-sponsored IND number: IND 76,042

Study Co-Chair
Elizabeth R. Gerstner, MD
Massachusetts General Hospital
The Stephen E. and Catherine Pappas Center for Neuro-Oncology
55 Fruit Street
Yawkey 9E
Boston, MA 02114
Phone: 617-724-2887
Fax: 617-643-2591
Email: egerstner@partners.org

Study Co-Chair
James R. Fink, MD
University of Washington Medical Center
Department of Radiology, Box 357115
1959 N. E. Pacific Street
Seattle, Washington 98195
Phone: 206-543-3320
Fax: 206-598-8475
Email: jrfink@u.washington.edu

Co-Chair
David Mankoff, MD, PhD
Seattle Cancer Care Alliance
Radiology
825 Eastlake Avenue East
G2-600
Seattle, WA 98109
Phone: 206-288-2173
Fax: 206-288-6556
Email: dam@u.washington.edu

Study Statistician
Zheng Zhang, PhD
Center for Statistical Sciences
Brown University
121 South Main Street, Box G-S121-7
Providence, RI 02912
Phone: 401-863-2578
Fax: 401-863-9182
Email: zzhang@stat.brown.edu

PARTIAL PROTOCOL—CONTACT ACRIN PROTOCOL DEVELOPMENT AND REGULATORY COMPLIANCE FOR A COMPLETE PROTOCOL

Original Date: **March 24, 2009**
Activation Date: **August 24, 2009**
Version Date: **January 24, 2012**
Includes Amendments 1–7

CONFIDENTIAL

This protocol was designed and developed by the American College of Radiology Imaging Network (ACRIN). It is intended to be used only in conjunction with institution-specific IRB approval for study entry. No other use or reproduction is authorized by ACRIN, nor does ACRIN assume any responsibility for unauthorized use of this protocol.

Table of Contents

Schema

1.0 Abstract 4

2.0 Background and Significance 4

3.0 Study Objectives/Specific Aims 7

4.0 Study Drug Information 8

5.0 Study Overview 11

6.0 Participant Selection/Eligibility Criteria 12

7.0 Site Selection 14

8.0 Data Management/Online Registration 15

9.0 Study Procedures 19

10.0 Image Guidelines 26

11.0 Well-Counter Calibration/Blood Sampling Guidelines 32

12.0 Adverse Events Reporting 33

13.0 Ethical Considerations 43

14.0 Conflict of Interest 44

15.0 Publication Policy 44

16.0 Institutional Monitoring and Audits 44

17.0 Statistical Considerations **REMOVED FROM WEB VERSION** 47

18.0 Tissue Specimen Collection for Biomarker Analysis 49

References 52

Appendix I: Informed Consent Form Template 55

Appendix II: Supplemental Materials Available Online 67

Appendix III: Well Counter Cross-Calibration Procedure 68

Appendix IV: Blood Sampling Procedure 70

Appendix V: Macdonald Radiographic Response Criteria 71

Attachment A: Report on Radiation Dose Estimates for PET and PET/CT 72

AMERICAN COLLEGE OF RADIOLOGY IMAGING NETWORK

ACRIN 6684

MULTICENTER, PHASE II ASSESSMENT OF TUMOR HYPOXIA IN GLIOBLASTOMA USING ^{18}F-FLUOROMISONIDAZOLE (FMISO) WITH PET AND MRI

SCHEMA

**ELIGIBILITY**<br>Patients with pathologically-confirmed newly diagnosed glioblastoma (WHO grade IV) who have residual disease, are older than 18 years, and scheduled for standard chemoradiotherapy alone or with an investigational agent.
↓
**VISIT 1 REGISTRATION/SCREENING**
↓
**VISIT 2 IMAGING: FMISO PET AND MRI SCANS**<br>(See Study Procedures Section 9.0 for details.)
↓
**VISIT 2A IMAGING: REPRODUCIBILITY FMISO PET SCAN**<br>A sub-set (15 participants) will undergo an additional FMISO PET scan prior to initiation of chemoradiation.
↓
**FOLLOW UP: UP TO 5 YEARS FOR PROGRESSION AND SURVIVORSHIP**<br>All standard of care MRIs and CTs will be submitted to ACRIN.

STUDY OBJECTIVES

To determine the association of baseline FMISO PET uptake (tumor to blood ratio, hypoxic volume) and MRI parameters (K^{trans}, CBV) with overall survival, time to disease progression, and 6-month progression free survival in participants with newly diagnosed glioblastoma (GBM).

ELIGIBILITY *(see Section 6.0 for details)*

Adult patients newly diagnosed with GBM (World Health Organization [WHO] grade IV) and visible residual disease planned for initial treatment with radiation therapy (RT) and temozolomide (TMZ), with or without additional agents, will be enrolled. Amount of residual tumor will not impact eligibility and visible residual disease can include T2/FLAIR hyperintensity.

SAMPLE SIZE

A minimum of 50 participants will be enrolled. A subset of 15 participants will have test-retest FMISO PET scans at baseline performed between 1 and 7 days apart (both scans completed prior to initiation of chemoradiation). The timeframe for recruitment will be approximately 1 year, and participants will be recruited from 4 to 10 sites. Each institution will not enroll more than 15 participants to this trial.

1.0 ABSTRACT

This protocol for human research study is conducted according to United States and international standards of Good Clinical Practice (International Conference on Harmonisation [ICH] Guidelines), applicable government regulations (e.g. Title 45, Part 46 Code of Federal Regulations) and the American College of Radiology Imaging Network (ACRIN) research policies and procedures.

Glioblastoma (GBM) is the most common and, unfortunately, most aggressive type of primary malignant brain tumor. Despite treatment with surgery, radiation, and temozolomide (TMZ) chemotherapy, median overall survival (OS) is less than 15 months.[1] One of the pathologic hallmarks of GBM is necrosis, thought to be the result of tumor hypoxia secondary to an inefficient blood supply. Hypoxia is a potent stimulator of angiogenesis, a requisite for tumor growth.[2] In addition, hypoxia limits the efficacy of radiation and chemotherapy and may select for a more aggressive tumor phenotype. By better understanding the extent of tumor hypoxia and changes in regional tumor hypoxia with treatment, more effective and patient-specific therapies could be devised to halt GBM growth. For example, patients with persistent areas of tumor hypoxia may benefit from adding therapies that improve oxygenation, are hypoxia-selective, or target downstream consequences of hypoxia, especially angiogenesis.

^{18}F-Fluoromisonidazole (FMISO) PET is a non-invasive method that can estimate tissue hypoxia. Several studies have validated FMISO uptake as a robust measure of tissue hypoxia, and methodology for FMISO PET imaging has been established in early single-center studies.[3-7] One preliminary study of 22 participants with GBM demonstrated an association with both the pre-radiation volume and degree of tumor hypoxia measured by FMISO PET and a shorter time to tumor progression and survival (Spence et al 2008).[3] Consequently, knowledge of the amount and distribution of tumor hypoxia may provide prognostic information as well as useful information to guide therapy for patients with GBM.

New advanced MRI methods, such as dynamic contrast enhanced (DCE) MRI, oxygen breathing during BOLD imaging, vessel caliber mapping, or other approaches may also contribute to characterization of tumor physiology and/or prognostic information for patients with GBM. Combining such vascular-imaging methods with hypoxia data from FMISO uptake is very attractive. This also recognizes the mechanistic connection between hypoxia and angiogenesis and provides an opportunity to study the association of hypoxia, by FMISO PET, and tumor vascularity, by perfusion MRI. We propose using these PET and MRI techniques hand in hand to monitor the standard initial treatment of patients with GBM and to perform pilot work to determine if FMISO PET may be a useful prognostic marker in patients with GBM. Data gained from this study will also serve as the basis for future clinical trials where hypoxia imaging could be used for correlative science, patient selection, and response evaluation. Both MRI and PET imaging are low-risk tests so the potential knowledge gained about tumor biology in this study makes it a worthwhile endeavor.

2.0 BACKGROUND AND SIGNIFICANCE

Hypoxia is a potent stimulator of angiogenesis, a requisite for tumor growth. In addition, hypoxia limits the efficacy of radiation and chemotherapy and may select for a more aggressive tumor phenotype. By better understanding the extent of tumor hypoxia and changes in regional tumor hypoxia with treatment, more effective and patient-specific therapies could be devised to halt GBM growth. For example, patients with persistent areas of tumor hypoxia may benefit from

adding therapies that improve oxygenation, are hypoxia-selective, or target downstream consequences of hypoxia, especially angiogenesis.

2.1 FMISO PET as a Hypoxia Imaging Biomarker

Since the 1980s, interest in radiolabeled nitroimidazoles, and particularly FMISO, as nuclear imaging agents of hypoxia has been growing. Intracellular nitroreductases metabolize nitroimidazoles in the setting of low oxygen levels, thus trapping them in hypoxic cells. Importantly, the metabolism of nitroimidazoles relies on an active electron transport chain so only live cells can trap FMISO. The binding of FMISO is related to the level of hypoxia, and oxygen levels of 3 to 10 mm Hg lead to FMISO binding.[8,9] Several studies have demonstrated that the uptake of FMISO correlates well with invasive polarographic oxygen electrodes, the gold standard to determine tissue oxygenation.[10-13]

Preliminary studies of FMISO uptake in glioma have demonstrated the feasibility of this technique both in animal models and in humans.[6] In a glioma rat model, FMISO uptake was significantly greater in tumor tissue than in surrounding normal brain.[5] Cher et al showed that all high-grade gliomas have increased FMISO uptake while low-grade gliomas did not, which is consistent with the underlying biology of gliomas—higher-grade tumors are more hypoxic than lower-grade tumors.[7] In 22 participants with newly-diagnosed GBM, the greater the volume and degree of hypoxia based on FMISO imaging, the shorter time-to-tumor progression and survival.[3] In addition, histological studies of tumors have correlated tissue pimonidazole staining, a marker of tissue hypoxia, and CAIX with FMISO uptake.[14,15] In one of these studies, the glioma model had the strongest correlation between FMISO uptake and pimonidazole staining.[14] *Ex vivo* autoradiography has also been used to confirm the correlation of FMISO and tumor hypoxia.[16]

2.2 Tissue:Blood or Tissue:Cerebellum

The most predictive FMISO imaging markers for viewing these hypoxic distinctions have derived from tissue:blood FMISO concentration ratios, which compare image measures of tissue uptake concentration to blood concentration levels measured by blood sampling. The Spence study[3] showed that markers established by prior studies,[12] namely the maximum tumor:blood ratio in the tumor site (T/B_{max}) and the hypoxic volume (HV, volume of tumor tissue with tissue:blood > 1.2), were predictive of patient survival.

FMISO uptake is currently quantified using tissue:blood (T/B) ratios obtained from comparing image measures of tissue uptake to blood concentration from blood samples. This approach has been extensively validated against "gold standard" measures of hypoxia[8,12] and has shown to be predictive of survival in GBM.[3] However, this approach requires blood sampling and cross-calibration between the PET scanner and the well counter used to measure blood concentration. FMISO is highly lipophilic and fairly uniformly distributed in normal, non-hypoxic brain tissue; therefore, normal brain uptake may provide a reasonable proxy for blood concentration. Preliminary data from Dr. Spence's group have shown a high degree of correlation between FMISO uptake ratios using the normal cerebellum as a reference region and indirect measure of blood concentration (T/C ratio) versus T/B. As a secondary objective of this study, we will correlate T/C and T/B as measures of FMISO uptake and test the predictive value of T/C. If T/C is shown to be equivalent to T/B, this would enable future use of FMISO by imaging only, without blood samples, and eliminate the need for cross-calibration of the

PET scanner with a well counter. This would significantly enhance feasibility of FMISO PET in future clinical trials.

Interestingly, a study of 11 participants with various brain tumors that combined hypoxia measurement using FMISO and perfusion measurement using $^{15}O\text{-}H_20$ demonstrated that low perfusion was not necessarily associated with hypoxia.[4] The reason for this discrepancy likely reflects the complicated relationship in gliomas between perfusion and hypoxia. Recent data suggest that tumor tissue hypoxia may cycle with a periodicity of 20 to 30 minutes, which may explain some of these findings .[2] Other data have suggested that, at least in some tumors, FMISO uptake correlates with perfusion measures.[17] The ability of MRI to assess perfusion and other hemodynamic properties of tumors makes it an ideal tool to combine with FMISO PET.

2.3 Viewing Vasculature: MRI Complements FMISO PET

MRI provides functional information as well as anatomical information about tumor vasculature. Cerebral blood flow, blood volume, and vessel size can be used to determine if a mismatch exists between tumor vascular function and hypoxia because these physiologic parameters accurately reflect the heterogeneous nature of gliomas. Changes in flow, vascular density, and/or the geometry of microvessels are all thought to regulate tumor oxygenation[2] and can be measured with dynamic susceptibility MRI. Vascular permeability as measured by K^{trans} (the vascular transfer constant) or Ve (the volume of extravascular extracellular space) would provide further information about vessel function and the role that elevated interstitial fluid pressure plays in hypoxia.

In one animal model of colorectal cancer, reduction in K^{trans} and Ve correlated with an increase in oxygenation following radiation.[18] In addition, breathing oxygen or carbogen during BOLD MRI has been shown to reveal dynamic changes in tumor vascular physiology.[19,20] Therefore, the complementary information provided by physiological MRI parameters and metabolic information provided by FMISO PET will help to clarify the discrepancy between perfusion and hypoxia, which has significant clinical implications.

Being able to better characterize and target the mechanisms of GBM hypoxia (e.g. hypoperfusion) could lead to improvements in therapy for this deadly disease. There are approximately 10,000 GBMs diagnosed each year in the United States and the median overall survival is less than 15 months. Consequently, better therapies are desperately needed. Considering that the standard treatment for GBM—radiation and TMZ chemotherapy—relies on adequate oxygenation to be maximally effective, a better understanding of tumor hypoxia and perfusion could optimize these therapies and improve survival.

Furthermore, if FMISO becomes a clinically valuable tool, measurement methods such as tumor:cerebellum ratios would be more convenient to use than the current practice of blood sampling and cross calibration. Hence, in this study we will seek to determine the feasibility of the tumor:cerebellum method compared with tumor:blood as we both elucidate the presence and mechanisms of hypoxia and assess the robustness of hypoxia imaging with FMISO PET.

2.4 Justification and Overall Goals of the ACRIN 6684 FMISO PET Trial

This protocol is a multi-center, phase II trial to assess tumor hypoxia in GMB using FMISO with PET and MRI. In the initial data by Spence et al, FMISO was a useful

prognostic marker.[3] However, this was a small single-centered trial so the data will need to be confirmed in a multicenter trial.

For ACRIN 6684, the role of imaging in this trial is primarily prognostic. The overall goals of the study are both clinical and biological. The primary aim is to establish hypoxia imaging as a tool to select more aggressive and/or hypoxia-targeted therapy in particular subsets of patients.

2.5 Impact of Including Additional Therapeutic Agents

The primary goal of this study is to determine the prognostic value of FMISO PET prior to start of treatment. Therefore, allowing all available therapeutic options for trial participants following the FMISO PET does not impact our primary endpoint, which, again, looks at the association of baseline hypoxic volume and overall survival. Patients with GBM are treated with a variety of therapies, so limiting eligibility to standard chemoradiation alone or only with the addition of anti-VEGF treatment or an anti-PARP inhibitor is restrictive and unnecessary. Removal of the restrictive therapy-related eligibility criteria introduced in earlier amendments to the protocol (revised to be more all-inclusive therapeutically as of Amendment 7) will speed study accrual and increase the likelihood of trial completion.

3.0 STUDY OBJECTIVES/SPECIFIC AIMS

3.1 Primary Aim

To determine the association of **baseline** FMISO PET uptake (hypoxic volume [HV], highest tumor:blood ratio [T/B_{max}]) and MRI parameters (K^{trans}, CBV) with **overall survival (OS)** in participants with newly diagnosed GBM.

3.2 Secondary Aims

3.2.1 **Aim 1:** To determine the association of **baseline** FMISO PET uptake (HV, T/B_{max}) and MRI parameters (K^{trans}, CBV) with **time to progression (TTP)** and **6-month progression free survival (PFS-6)** in participants with newly diagnosed GBM.

3.2.2 **Aim 2:** To assess the **reproducibility** of the **baseline** FMISO PET uptake parameters by implementing baseline **"test"** and **"retest" PET scans** (performed within 1 to 7 days of each other).

3.2.3 **Aim 3:** To assess the **correlation** between **highest tissue:cerebellum ratio** [T/C_{max}] and **T/B_{max}** at **baseline**.

3.2.4 **Aim 4:** To assess the **correlation** between **other MRI parameters** (T1Gd, VCI, CBV-S, ADC, NAA-Cho, BOLD, T2) and **OS, TTP,** and **PFS-6**.

4.0 STUDY DRUG INFORMATION

4.1 Description of ^{18}F-fluoromisonidazole (FMISO) (NSC #742836; IND # 76,042)

The Investigator Brochure for FMISO will be available to ACRIN-approved sites. The brochure will provide detailed information regarding the investigational agent. For adverse events related to this agent, see Section 13.0.

4.1.1 *Chemical Name*: 1H-1-(3-[^{18}F]-fluoro-2-hydroxy-propyl)-2-nitro-imidazole

4.1.2 *Other Name:* [^{18}F]-fluoromisonidazole, [^{18}F]FMISO, FMISO

4.1.3 *Molecular Formula:*

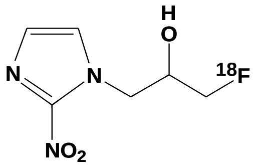

4.1.4 *Description:* [^{18}F]-fluoromisonidazole ([^{18}F]FMISO, FMISO) is a radiolabeled imaging agent that has been used for investigating tumor hypoxia with PET. FMISO is an azomycin-based hypoxic cell sensitizer that binds covalently to cellular molecules at rates that are inversely proportional to intracellular oxygen concentration, rather than by any downstream biochemical interactions. In hypoxic cells, FMISO is trapped, which is the basis for the use of this tracer to measure hypoxia. The physical half-life of FMISO is 110 minutes.

4.1.5 *Supplier/How Supplied:*

FMISO is available under the National Cancer Institute (NCI), Cancer Imaging Program's (CIP's) Investigational New Drug Application (IND) filed with the Food and Drug Administration (FDA). FMISO is supplied by Cardinal Health, which has previously provided NCI with a letter of authorization to cross-reference their Drug Master File filed with the FDA.

Local sites may make their own FMISO only if their chemistry, manufacturing, and control (CMC) processes and standard operating procedures (SOPs) have already been filed within the NCI IND and have met all requirements in accordance with FDA regulations and guidance.

The radiopharmaceutical product, FMISO, is the only active ingredient and it is dissolved in a solution of ≤10 mL of 95% isotonic saline: 5% ethanol (v:v). FMISO is provided as a ready to use isotonic, sterile, pyrogen-free, clear, and colorless solution. FMISO is typically packaged in a glass vial and does not contain any preservatives.

4.1.6 *Dosage and Route of Administration:* FMISO is administered to subjects by intravenous injection by bolus of ≤10 mL. The FMISO dose for this protocol should be 3.7 MBq/kg (0.1 mCi/kg) up to a maximum of 260 MBq (7 mCi).

4.1.7 *Storage:* FMISO should be stored upright in an appropriate lead or tungsten alloy shielded container at room temperature.

4.1.8 *Stability:* Refer to the guidelines from the provider, but typically FMISO should be used within 8-12 hours of the end of synthesis.

4.1.9 *Precaution:* The use of FMISO is limited to experimental use for diagnostic imaging studies. FMISO should be administered by nuclear medicine personnel trained to handle radioactive material.

4.1.10 *Synthesis, Apyrogenicity, and Purity:* FMISO will be synthesized and tested following the procedures filed in IND 76,042 by any site with full CMC data filed in the IND or supplied by Cardinal Health from any site that is filed under the Cardinal DMF, which is referenced in IND 76,042. All manufacturing and testing comply with USP<823>. The apyrogenicity of the product will be established with the bacterial endotoxin test. The radiochemical purity of the product will be evaluated by high-performance liquid chromatography and/or thin-layer chromatography (TLC) and is 95% or greater.

4.2 Supplier of FMISO

FMISO is supplied by Cardinal Health or is manufactured on site by any site with full Chemistry, Manufacturing, and Controls (CMC) data filed in the IND.

4.2.1 *Agent Ordering:* Sites approved to manufacture their own FMISO may do so for this study after providing CMC data to ACRIN regulatory personnel.

Sites who will order the FMISO from Cardinal Health will need to complete a study drug request form. Instructions for request and completion of the request form will be made available to those sites.

4.2.2 *Agent Returns:* Not applicable. Unused drug should be allowed to decay and disposed of in accordance with institution guidelines.

4.2.3 *Agent Accountability:* The investigator or the investigator-designee must maintain a detailed record of:

4.2.3.1 Volume/Dose Received – Date, Time, By whom

4.2.3.2 Volume/Dose Transfer – Date, Time, From/To whom

4.2.3.3 Volume/Dose Drawn – Date, Time, Method, By whom

4.2.3.4 Volume/Dose Administered – Date, Time, Subject ID, Administered by whom, Volume into subject, Volume remaining in administration device, Volume remaining in delivery container, Disposition of all UN-administered volumes & contaminated containers [may refer to use of standard facility protocol document/SOP if that document covers this detail, e.g. "Per (institution) SOP (#)"]

4.3 FMISO Biodistribution and Radiation Dosimetry

The radiation exposure from FMISO in this study will be equal to or lower than that of other widely used nuclear-medicine experimental research agents. Increased voiding frequency will reduce the radiation dose to the bladder wall, which is the organ site that

receives the highest radiation absorbed dose. Potential radiation-specific risks associated with this PET study are within generally accepted limits for such studies.

In the dose of FMISO for this study, only a small fraction of the FMISO molecules are radioactive. The amount of injected drug is ≤ 15 µg (≤ 80 nmol per dose) of FMISO. FMISO is administered to subjects by intravenous injection of ≤ 10 mL. There is no evidence that nonradioactive and radioactive FMISO molecules display different biochemical behavior.

Table 1. Radiation absorbed dose to organs for standard man

Tissue	Mean (mGy/MBq)	Mean (mrad/mCi)	Total / 7 mCi (mrad)
adrenals	0.0166	61.4	430
brain	0.0086	31.8	223
breasts	0.0123	45.5	319
gall bladder wall	0.0148	54.8	383
lower large intestine	0.0143	52.9	370
small intestine	0.0132	48.8	342
stomach	0.0126	46.6	326
upper large intestine	0.0140	51.8	363
heart wall	0.0185	68.5	479
kidneys	0.0157	58.1	407
liver	0.0183	67.7	474
lungs	0.0099	36.6	256
muscle	0.0142	52.5	368
ovaries	0.0176	65.1	456
pancreas	0.0179	66.2	464
red marrow	0.0109	40.3	282
bone surface	0.0077	28.5	199
skin	0.0048	17.8	124
spleen	0.0163	60.3	422
testes	0.0146	54.0	378
thymus	0.0155	57.4	401
thyroid	0.0151	55.9	391
**urinary bladder wall**	0.0210	77.7	544
uterus	0.0183	67.7	474
eye lens	0.0154	57.0	399
Total body	0.0126	46.6	325

(**Source:** [^{18}F]FMISO Investigators Brochure)

Calculated total body dose for a 70 kg man injected with 3.7 MBq/kg was 0.013 mGy/MBq; for a 57 Kg woman it was 0.016 mGy/MBq. Effective dose equivalents (EDE) were 0.013 mSv/MBq for men and 0.014 mSv/MBq for women. Ninety-seven percent of the injected radiation was homogenously distributed in the body, leaving only 3% for urinary excretion. Doses to smaller organs not directly determined by visualization, such as the lens, were calculated assuming average total-body concentrations. The absence of tracer visualized in images of those organs indicated that

accumulation there was not increased. Expected EDE for a 56-kg female is 3.0 mSv (300 mRem) and for a 70-kg male is 3.4 mSv (340 mRem).

More recently, radiation exposure for radiopharmaceuticals has been expressed as the effective dose (ED). The estimated ED for [^{18}F]FMISO is 0.015 mSv/MBq (see Table 1 in Attachment A to the protocol). Therefore, for the maximum 7 mCi dose, the maximum emission exposure is 3.9 mSv.

4.4 Monitoring for Physiologic Effects of FMISO

4.4.1 *Vital signs:*

Vital signs, including temperature, blood pressure, heart rate, and respiratory rate, will be measured prior to injection and at completion of FMISO PET imaging, and subsequently as needed.

4.4.2 *Laboratory studies:*

No routine laboratory studies are required to monitor FMISO use, but this patient population will have weekly complete blood counts and frequent serum chemistry as part of routine clinical care. These data will not be collected for the study.

4.5 Monitoring for Adverse Events

Adverse events (AEs) will be evaluated at each imaging session; AE monitoring will cover at least ten half-lives of the FMISO drug, or 24 hours. AEs for FMISO are defined as any signs of illness or symptoms that have appeared or worsened since the infusion of the FMISO. Participants will be queried for potential AEs:

- At the time of injection;
- Before leaving the PET suite;
- If they call the site as instructed for any concerns during the 24 hours after FMISO administration;
- By telephone after 24 hours post-FMISO infusion.

The AEs that will be specifically monitored during and after the infusion include: localized discomfort at the intravenous (IV) injection site, pain, respiratory difficulties, blood pressure instability, flushing, dizziness, pruritis/rash, and any other symptoms that could be related to an allergic or anaphylactoid-type reaction. When an AE is reported, concomitant medication taken by the participant in the 2 weeks prior to the event and/or during the time of the AE will be collected and documented. (See Section 12.0 for AE reporting requirements.)

5.0 STUDY OVERVIEW

A minimum of 50 patients with newly diagnosed GBM will be recruited. The trial will continue until accrual meets the target of 50 eligible and evaluable participants. “Evaluable” is defined as: images are completed; site deems all images adequate; and images pass initial quality review at ACRIN PET and MRI Core Labs. Inevaluable and ineligible cases will be followed per protocol (see Section 9.3). Although some visible disease is required for entry, the amount of residual tumor after surgery will not impact eligibility and visible residual disease can include T2/FLAIR hyperintensity. The timeframe for recruitment will be approximately 1 year, and participants will be recruited from 4 to 10 sites. Each institution will not enroll more than 15 participants to this trial. The subset of 15 participants in the test-retest substudy will have consented to a second baseline FMISO PET scan within 7 days of the first scan; if only one scan is completed for any

reason prior to treatment initiation, then additional participants will need to be recruited until 15 test-retest sets have been completed. All participants will be asked to complete the test-retest substudy until 15 participants have completed the series. After the test-retest substudy has been completed, potential participants will be consented only to the baseline scan. Focus will be on completing the baseline FMISO PET and MRI scans, and trial accrual will continue to reach targeted goals even if the test-retest substudy is incomplete.

In this study, participants with residual GBM will undergo FMISO PET and MRI scans after surgery and prior to start of chemoradiation (baseline). The subset of 15 participants will have two FMISO PET scans within 1 to 7 days of each other to test reproducibility of FMISO PET; this substudy will continue to accrue until 15 participants have completed the two test-retest FMISO PET scans. Participants will then resume standard of care imaging for GBM per treating physician discretion, which will be submitted to ACRIN. Participants will be followed until death or the end of the study.

6.0 PARTICIPANT SELECTION/ELIGIBILITY CRITERIA

Adult patients who have residual disease after surgical removal of newly-diagnosed GBM, based on pathological confirmation, will be eligible for this trial. Additional therapies to temozolomide and radiation are allowable; ACRIN requests information about scheduled concomitant therapy at registration). Should the concomitant agent be available through another clinical trial, the site will need to ensure treatment details (dose, time of administration, and any interruptions in therapy) are available to ACRIN. See Appendix VI for a list of example concomitant agents allowable for this trial.

6.1 Inclusion Criteria

6.1.1 Must be able to provide a written informed consent;

6.1.2 Must be 18 years or older;

6.1.3 Newly diagnosed GBM, WHO grade IV based on pathology confirmation;

6.1.4 Residual tumor after surgery (amount of residual tumor will not impact patient eligibility and visible residual disease can include T2/FLAIR hyperintensity);

NOTE: If patient had a biopsy only, postoperative MRI is not needed to assess residual tumor prior to enrollment.

6.1.5 Scheduled to receive standard fractionated radiation therapy;

6.1.6 Scheduled to receive TMZ in addition to radiation therapy;

6.1.7 Karnofsky Performance Score > 60.

6.2 Exclusion Criteria

6.2.1 Pregnant or breastfeeding (if a female is of child-bearing potential, and unsure of pregnancy status, a standard urine pregnancy test should be done);

6.2.2 Scheduled to receive chemotherapy, immunotherapy, or investigational agents in trials unwilling to share data with ACRIN (i.e., additional therapy added to radiation and TMZ is allowed if ACRIN is able to obtain treatment information);

6.2.3 Not suitable to undergo MRI or use the contrast agent Gd because of:

6.2.3.1 Claustrophobia;

6.2.3.2 Presence of metallic objects or implanted medical devices in body (i.e., cardiac pacemaker, aneurysm clips, surgical clips, prostheses, artificial hearts, valves with steel parts, metal fragments, shrapnel, tattoos near the eye, or steel implants);

6.2.3.3 Sickle cell disease;

6.2.3.4 Renal failure;

6.2.3.5 Reduced renal function, as determined by GFR < 30 mL/min/1.73 m^2 based on a serum creatinine level obtained within 28 days prior to registration;

6.2.4 Presence of any other co-existing condition which, in the judgment of the investigator, might increase the risk to the subject;

6.2.5 Presence of serious systemic illness, including: uncontrolled intercurrent infection, uncontrolled malignancy, significant renal disease, or psychiatric/social situations which might impact the survival endpoint of the study or limit compliance with study requirements;

6.2.6 History of allergic reactions attributed to compounds of similar chemical or biologic composition to FMISO. An allergic reaction to nitroimidazoles is highly unlikely;

6.2.7 Not suitable to undergo PET or MRI, including weight greater than 350 lbs (the weight limit for the MRI and PET table).

6.2.8 Prior treatment with implanted radiotherapy or chemotherapy sources such as wafers of polifeprosan 20 with carmustine.

6.3 Recruitment and Screening

Each institution will not enroll more than 15 participants to this trial. Potential participants will be referred by their treating neuro-oncologists or medical oncologists, who will be made aware of the study by the site principal investigator (PI). Potential participants will be approached and consented by a member of the study team.

ACRIN will allow patients to join this trial who are enrolled also in trials using additional investigational agents even if the trial is blinded. However, ACRIN will eventually need to access unblinded treatment and regimen data (specifically, ACRIN will need to know details of the timing, dosing, and any interruption of the therapeutic regimen). The site and ACRIN may need to coordinate with the other trial’s sponsorship to determine any necessary data sharing prior to patient enrollment to the ACRIN trial. ACRIN requests information about the participant’s scheduled concomitant therapy at time of registration.

Once eligibility criteria have been assessed and the informed consent is obtained, participants will undergo a screening process to further ensure eligibility. Screening prior to registration will comprise a review of pathology reports, postoperative MR images, operative reports, and medical history; general physical and neurologic exams; routine blood work; and urine pregnancy test for women of childbearing age.

ACRIN will develop a trial communications plan that will describe the production of materials to aid participant recruitment. All materials used for participant recruitment will be reviewed and approved by each institution's Institutional Review Board (IRB).

6.4 Inclusion of Women and Minorities

Both men and women and members of all ethnic groups are eligible for this trial. In conformance with the National Institutes of Health (NIH) Revitalization Act of 1993, with regard to inclusion of women and minorities in clinical research, the projected gender and minority accruals are shown below in Table 2:

Table 2. Gender and Minority Accrual Estimates

Ethnic Category	Sex/Gender			
	Females	Males	Unknown	Total
Hispanic or Latino	4	5	0	9
Not Hispanic or Latino	17	24	0	41
**Ethnic Category: Total of all subjects**	**21**	**29**	0	**50**
**Racial Category**				
American Indian or Alaskan Native	0	0	0	0
Asian	3	4	0	7
Black or African American	4	5	0	9
Native Hawaiian or other Pacific Islander	0	0	0	0
White	14	20	0	34
**Racial Category: Total of all subjects**	**21**	**29**	0	**50**

7.0 SITE SELECTION

7.1 Institution Requirements

The potential sites for this study are ACRIN-approved institutions that also meet study-specific qualifications. If a site previously has not been approved to participate in ACRIN clinical trials, a General Qualifying Application (GQA) must be submitted. In addition, each institution must submit a Protocol Specific Application (PSA). The applications and form are available at www.acrin.org/6684_protocol.aspx.

Potential site must have access to local or commercial supply of the FMISO agent according to the parameters of the NCI-sponsored IND. This will ensure that the FMISO tracer arrives successfully to the site and can be handled appropriately. For any sites approved to manufacture their own FMISO, the site must provide CMC data to ACRIN regulatory personnel as part of the site qualification process.

In addition, the sites will need to have a well counter appropriate for counting blood samples and providing estimates of radioactivity concentrations that are cross-calibrated to PET scanner estimates of radioactivity concentrations in the PET images; phantom and calibration procedures will need to be run as a pre-qualifier for the well calibration procedure (see Section 11.0, Appendix III, and www.acrin.org/6684_imagingmaterials.aspx for details).

Sites must submit and pass a series of qualification steps, including a test FMISO PET scan and a test MRI/MRS scan per protocol specifications (see Section 10.0) prior to being qualified to participate. Sites must also have PET scanners and MR images approved prior to the institution participating in the study. All scanner and image qualification materials are available at www.acrin.org/6684_protocol.aspx. Section 10.0 provides detailed information regarding scanning equipment and imaging-procedure protocols. All regulatory documentation must be submitted to ACRIN Headquarters (via fax: 215-717-0936, ATTN: ACRIN 6684 Project Manager).

7.2 Form FDA 1572, IRB Approval Letter, and Informed Consent Form

All institutions must submit study-specific Institutional Review Board (IRB) approval for the protocol and informed consent form (ICF), along with a Form FDA 1572. (The ICF is included in this protocol as Appendix I). The investigator and the investigator-designated research staff must follow OHRP-approved consent procedures (Title 45, Part 46 Code of Federal Regulations), as well as those set by the local IRB at the institution. Prior to registering the first participant, the completed Form FDA 1572, a copy of the IRB approval letter, and a copy of the IRB-approved, site-specific ICF must be delivered to the trial monitor for review and to be kept on file at ACRIN Headquarters (fax: 215-717-0936, ATTN: Protocol Development and Regulatory Compliance Department).

7.3 Accrual Goals and Monitoring

The ACRIN Biostatistics and Data Management Center (BDMC) will monitor participant accrual. Total target accrual for this study is 50 participants within a year. The trial will continue until accrual meets the target of 50 eligible and evaluable participants. “Evaluable” is defined as: images are completed; site deems all images adequate; and images pass initial quality review at ACRIN PET and MRI Core Labs. Inevaluable and ineligible cases will be followed per protocol (see Section 9.3). Each institution will not enroll more than 15 participants to this trial. During the recruitment period, accrual will be reviewed monthly with the intention of discovering and resolving any recruitment barriers. In particular, starting approximately one month after a site is approved to begin participant enrollment, the site’s actual accrual will be compared to the average monthly accrual potential described in their PSA. If a site’s actual accrual falls significantly below what is reported in their PSA, the Protocol Support Enrollment Committee (PSEC) comprised of the trial PI and his or her designees will determine a follow-up action plan to identify site accrual barriers and develop strategies to support the site in meeting accrual goals.

The ACRIN Steering Committee regularly reviews the overall trial accrual and may request information about a trial’s accrual performance to better understand general accrual barriers or issues. Accrual and safety information will be presented to the ACRIN Data and Safety Monitoring Committee (DSMC) at regularly scheduled meetings thereof; the DSMC may, at its discretion, re-evaluate the study with respect to feasibility or the need for additional participating institutions.

8.0 DATA MANAGEMENT/ONLINE REGISTRATION

8.1 General

8.1.1 The ACRIN web address is www.acrin.org**.**

8.1.2 Data collection and management will be performed by the Biostatistics and Data Management Center (BDMC) of ACRIN under the direction of Dr. Constantine Gatsonis. The Biostatistics Center (BC) is located at Center for Statistical Sciences at Brown University in Providence, RI, and the Data Management Center (DMC) is located at ACRIN in Philadelphia, PA.

8.1.3 Participant enrollment and data collection occurs through a series of programmed screens accessed through the ACRIN web site to register/randomize participants, collect participant data, and maintain calendars of data submissions for each participant. By using the World Wide Web, ACRIN has made participant registration, data entry, and updated calendar information available to clinical sites 24 hours a day, seven days a week. Each successful case registration is confirmed through receipt of an e-mail containing a registration/randomization confirmation and a case specific calendar identifying timelines for data and image submission. If the confirmation e-mail is not received, the enrolling person should contact the DMC before attempting a re-registration. A DMC contact list is located on the ACRIN web site for each protocol.

8.2 Clinical Data Submission

8.2.1 Upon successful participant registration, a confirmation e-mail containing the registration and case specific calendar is sent to the research staff enrolling the participant via the web. In addition, the investigator-designated research staff may download the participant specific data submission calendar, which lists all forms and designated reports required by protocol, along with the form due dates at the DMC. These calendars will be updated as the study proceeds to reflect data that have been received, reply deadlines for queries about unclear data, deadlines for follow-up reports of adverse events, or changes in the protocol that change the data being collected or the timeframe. Updated calendars for each participant can be obtained 24 hours a day from the ACRIN web site. The research associate may use the calendar as a case management tool for data submission and follow-up scheduling.

8.2.2 The investigative site is required to submit data according to protocol as detailed on each participant’s calendar, as long as the case status is designated as open/alive or until the study is terminated. The case is closed when all data have been received, reviewed, and no outstanding data query exists for the case.

8.2.3 To submit data via the ACRIN web site, the appropriate investigator-designated research staff will log onto the ACRIN web site and supply the pre-assigned user name and password. Case report forms will be available on the web site through a series of links. Each web form is separated into modules; each module must be completed sequentially in order for the internal programming to be accurate. The user selects the link to the appropriate form and enters data directly into the web-based form. As information is entered into the web form application, various logic checks will be performed. These logic checks look for data that are missing, data that are out of range, and data that are in the wrong format (e.g. character data in a field requiring numeric responses). Such errors will be detected as soon as the user attempts to either submit the form or move

to the next data element. They must be corrected before the form is transmitted to the DMC. The user will not be able to finalize form transmission to the DMC until all data entered pass these logic checks. Forms that are not completed in one sitting can still be submitted and completed at a later date. The form will remain available on the web until the "Complete Form Submission" button is depressed.

8.2.4 Once data entry of a form is complete, and the summary form is reviewed for completeness and accuracy, the investigator or the research staff presses the "Complete Form Submission" button on the form summary screen and the data are transferred into the clinical database. No further direct revision of the submitted data are allowed after this point. E-mail confirmation of web data entry is automatically generated and sent to the site investigator or research associate listing all of the data generated and just submitted. Should a problem occur during transmission and the e-mail confirmation of data submission is not received, the investigator or research associate should contact the DMC for resolution of the submission.

8.2.5 If a temporary problem prevents access to the Internet, all sites are notified of the event and estimated down time through an ACRIN broadcast message. The investigative site should wait until access is restored to submit data. If the site discovers a problem with access to the Internet, the site RA or investigator should notify the DMC of the problem and the DMC will give an estimated time when access will be restored. If access will be unavailable for an extended period, sites must seek another Internet Service Provider (ISP). On a short-term basis, the ACR can serve as an ISP.

8.3 Data Security

The registration and data collection system has a built-in security feature that encrypts all data for transmission in both directions, preventing unauthorized access to confidential participant information. Access to the system will be controlled by a sequence of identification codes and passwords.

8.4 Electronic Data Management

8.4.1 Data received from the web-based forms are electronically stamped with the date and time of receipt by the ACRIN server. The data are then entered into the database. A protocol-specific validation program is used to perform more extensive data checks for accuracy and completeness. Complementary validation programs are initiated at the Brown BC and the DMC. The logic checks performed on the data at this point are more comprehensive than those built into the web-based data entry screens. They include checking that answers are logical, based on data entered earlier in the current form and the more thorough checks. Data elements that fail validation are followed up by the DMC research associate. The validation program generated by BC produces a log of errors, which is sent to the DMC for resolution. The program is frequently updated to incorporate exceptions to rules so that subsequent validity checks minimize the time the DMC needs to spend resolving problems. Additional data review will take place once the data are transferred to the BC. The BC will run

thorough cross-form validations, frequency distributions to look for unexpected patterns in data, and other summaries needed for study monitoring. Any errors found at the BC will be reported to the DMC for resolution. All BDMC communication with the participating sites is normally done through the DMC.

8.4.2 If checks at DMC or BC detect missing or problematic data, the DMC personnel assigned to the protocol sends a Request for Information (Z1 query letter) to the site RA or investigator specifying the problem and requesting clarification. The DMC updates the participant's data submission calendar with the due date for the site RA or investigator's response.

8.5 Missing and Delinquent Data Submission

In addition to providing the site with a data collection calendar for each case, the DMC periodically prompts institutions for timely submission of data through the use of a Forms Due Report. Distributed at intervals via the electronic mail system directly to both the RA and the investigator at each site, this report lists data items (e.g. forms, reports, and images) that are delinquent and those that will be due before the next report date. In addition to prompting clinicians to submit overdue data, the Forms Due Report helps to reconcile the DMC's case file with that of the RA and/or investigator. Future Due Forms Report may be sent on an as needed basis in addition to past due reports. The site investigator or RA may use the Forms Due and Future Due Reports as a case management tool.

8.6 Data Quality Assurance

8.6.1 The BC at Brown University will maintain a study database at its site for monitoring data quality and for performing analyses. These data are drawn directly from the permanent database of the DMC. The transfer of data between the DMC and the BC has been validated through a series of checks consisting of roundtrip data verification in which data are sent back and forth to verify that the sent data are equivalent to the received data. These checks are repeated at random intervals during the course of a given study. Any discrepancies and other data quality issues will be referred to DMC for resolution, since only the DMC can correct the data file. No changes to the data will be made at the BC.

8.6.2 Ongoing monitoring of data is performed to assess compliance with the protocol and to look for unforeseen trends that may be indicative of procedural differences among clinical sites. If patterns are discovered in the data that appear to arise from causes specific to an institution, the BDMC will apprise the ACRIN Headquarters and the site of the problem, and work with the site, along with ACRIN Protocol Development and Regulatory Compliance (PDRC) department, until the problem has been resolved. If the BDMC, along with the PDRC, cannot find a resolution to the problem, it will be brought to the ACRIN Quality Assurance (QA) Committee for further discussion and resolution.

8.6.3 In addition, the ACRIN QA Monitor will review case report forms and source documents at several different time points: after first few participants enrolled and during the conduct of the trial, including staff changes at the participating sites. In addition, the QA Monitor will review the initial and annual regulatory

documents and any revised regulatory documents. This monitoring process ensures protocol and regulatory compliance, participant's welfare and safety, and provides resources to sites for clarification to the protocol and guidance in completion of the case report forms.

9.0 STUDY PROCEDURES

The trial will continue until accrual meets the target of 50 eligible and evaluable participants. "Evaluable" is defined as: images are completed; site deems all images adequate; and images pass initial quality review at ACRIN PET and MRI Core Labs. Inevaluable and ineligible cases will be followed per protocol (see Section 9.3). Each institution will not enroll more than 15 participants to this trial. All participants will be asked to complete the test-retest substudy until 15 participants have completed the series; after the substudy has been completed, potential participants will be consented only to the baseline scan. Focus will be on completing the baseline FMISO PET and MRI scans, and trial accrual will continue to reach targeted goals even if the test-retest substudy is incomplete.

NOTE: All PET scans should be performed on the same ACRIN-qualified scanner; if this is not feasible, then the same model scanner should be consistently used for a participant throughout the trial if at all possible. All ACRIN-qualified scanners will need to be cross-calibrated to the well counter on a quarterly basis or as specified by the manufacturer.

A global time reference will need to be used to record and report timing of dose counting, injection times, imaging start time, blood sampling time, and blood counting time. It is important that this time source be accessible for all modalities used in the FMISO PET study (dose calibration, well counter, etc). A portable stop watch or other time piece accurate to at least a minute and preferably seconds is recommended.

NOTE: For this protocol, "PET" may comprise PET, PET/CT, or MR-PET. Any scanner considered for use for the trial will need to be ACRIN qualified and approved prior to use.

9.1 VISIT 1: Eligibility/Registration or Screening Log

At the registration visit, the potential participant will be screened and confirmed for eligibility by the appropriate study-team designee prior to formal electronic registration*:

- Obtain a signed IRB approved informed consent prior to conducting any study-related procedures.
- Screen and confirm eligibility which includes:
  - Review of the inclusion and exclusion criteria, pathology reports, postoperative MR images, operative reports, medical history,

    NOTE: If patient had a biopsy only, postoperative MRI is not needed to assess residual tumor prior to enrollment.
  - Conduct mental health assessment (including Karnofsky and Mini Mental State Exam [MMSE] scores if these scores have not been completed within 7 days prior to enrollment),
  - Perform general physical and neurologic exams including vital signs assessment, and

- Review routine laboratory studies, including creatinine levels for GFR if not assessed 28 days prior to registration.

- Conduct a standard urine pregnancy test if the participant is unsure of her pregnancy status.
- Report any scheduled therapy at time of registration.†
- Obtain and send tumor tissue specimen that was obtained for the diagnosis of GBM to ACRIN designated pathology laboratory, if feasible; specific collection instructions, shipment, and processing facility information will be provided at the initiation of the trial and on the ACRIN web site: www.acrin.org/6684_protocol.aspx. See Section 18.0 for tumor tissue specimen details.

***NOTE:** When possible, individuals who are evaluated for participation in this study but are not able/willing to participate should have a Screening Log completed detailing the reason for non-participation (e.g., participant refused to provide consent, patient is ineligible, patient is enrolled on a competing trial, etc.).

†NOTE: ACRIN will allow patients to join this trial who are enrolled also in trials using additional investigational agents where the treatment given is randomized and initial assessment is blinded. However, ACRIN will eventually need to access unblinded treatment and regimen data (specifically, ACRIN will need to know details of the timing, dosing, and any interruption of the therapeutic regimen). The site and ACRIN may need to coordinate with the other trial's sponsorship to determine any necessary data sharing prior to patient enrollment to the ACRIN trial.

9.2 VISIT 2: Baseline—Pre-Treatment FMISO PET and Brain MRI With Contrast (Within 2 Weeks Prior to Initiation of Chemoradiation)

Visit 2 will occur between 1 to 14 days prior to initiation of chemoradiation. Participants MUST be scanned using the same ACRIN-approved MRI and PET scanners used for trial qualification, using the same protocol-specific parameters consistently at each time point.

NOTE: The contrast agents do not interact, so administration of FMISO followed by MRI within the 110 minutes prior to initiation of PET scan is an acceptable approach to same-day imaging.

- Assess participant for Karnofsky and MMSE scores (if not completed within 7 days of scan).
- Conduct a pregnancy test per the institution's standard of care if a participant is unsure of her pregnancy status.
- Evaluate creatinine levels for GFR (via participant record review or special testing if necessary) if not assessed within 28 days prior to Visit 2 MRI scan.
- Obtain vital signs before FMISO administration and after completion of imaging.
- Place two (2) IV catheters into the participant's arm veins, one IV catheter in each arm; one IV will be used to inject the bolus of FMISO, the other will be used for blood extraction. Blood must be drawn from the IV that was not used for the FMISO injection. For the FMISO injection, minimize the length of the IV tubing between the injection site and the vein to avoid FMISO being left in the tube.

- Perform a saline flush following the FMISO injection.
- Obtain blood samples at three (3) time points at 5-minute intervals (± 2 minutes) during the FMISO PET scan; see Appendix IV and www.acrin.org/6684_imagingmaterials.aspx for details.

  NOTE: The first sample will be taken at 5 minutes (± 2 minutes) after the initiation of the emission scan. ***Exact time of collection is key for this protocol.*** Time will need to be recorded from a global resource to be used throughout the study. Minutes and seconds will be recorded and reported.
- QC the well counter (see Appendix III and www.acrin.org/6684_imagingmaterials.aspx for details).
- Conduct same-day blood sample analysis; see Appendix IV for details.

  NOTE: ***Must note exact time of analysis (minutes and seconds preferred) using study's global time resource for reporting purposes.***
- Perform the FMISO PET scan per the imaging parameters described in this protocol (see Section 10.0 and www.acrin.org/6684_imagingmaterials.aspx for imaging specifics).
- Obtain steroid use/dose information prior to MRI.
- Perform a brain MRI scan with Gd contrast per the imaging parameters described in this protocol (see Section 10.0 and www.acrin.org/6684_imagingmaterials.aspx for imaging specifics). **OPTIONAL BOLD SEQUENCE:** Note that 0_2 administration and collection of 0_2 saturation levels are necessary with the BOLD MRI. Sites are requested to administer oxygen per site preference, at a minimum of 7 L per minute via nasal canula. If the participant is unable to undergo the administration of oxygen for a period of less than 10 minutes (e.g., because of a history of chronic obstructive pulmonary disease), then the BOLD imaging sequence should not be conducted.
- Query participants for potential AEs. When an AE is reported, collect information about concomitant medication taken in the 2 weeks prior to the event and/or during the time of any reported AE. AE queries should be conducted by appropriate study-staff designee at the following time points:
  - At the time of injection;
  - Before leaving the PET suite;
  - If they call the site as instructed for any concerns during the 24 hours after FMISO administration;
  - By telephone after 24 hours post-FMISO infusion.

NOTE: Participant disease, preference, or scheduling needs may necessitate two days of imaging to complete the FMISO PET and MRI scans; however, each two-day imaging cycle will increase the participant's risk because an additional IV catheter will be necessary. This risk should be discussed with the participant. If two days of imaging are necessary, then both scans must be completed during the appropriate divergent period for each Visit (in this case, within that 1 to 14 day period prior to initiation of chemoradiation).

9.2.1 VISIT 2A: SUBSET OF 15 PARTICIPANTS ONLY – Reproducibility Arm for FMISO PET (1 to 7 Days After Visit 2, Prior to Initiation of Chemoradiation)

For the subset of 15 participants who enroll in the test-retest substudy, Visit 2A will occur within 1 to 7 days after completion of Visit 2 but prior to the start of therapy in order to determine the reproducibility of FMISO PET scan results. Participants MUST be scanned using the same ACRIN-approved PET scanner used for trial qualification, using the same protocol-specific parameters consistently at each time point.

- Conduct a pregnancy test per the institution's standard of care if a participant is unsure of her pregnancy status.
- Obtain vital signs before FMISO administration and after completion of imaging.
- Place two (2) IV catheters into the participant's arm veins, one IV catheter in each arm; one IV will be used to inject the bolus of FMISO, the other will be used for blood extraction. Blood must be drawn from the IV that was not used for the FMISO injection. For the FMISO injection, minimize the length of the IV tubing between the injection site and the vein to avoid FMISO being left in the tube.
- Perform saline flush following the FMISO injection.
- Obtain blood samples at three (3) time points at 5-minute intervals (± 2 minutes) during the FMISO PET scan; see Appendix IV and www.acrin.org/6684_imagingmaterials.aspx for details.

  NOTE: The first sample will be taken at 5 minutes (± 2 minutes) after the initiation of the emission scan. ***Exact time of collection is key for this protocol.*** Time will need to be recorded from a global resource to be used throughout the study. Minutes and seconds will be recorded and reported.
- QC the well counter (see Appendix III and www.acrin.org/6684_imagingmaterials.aspx for details).
- Conduct same-day blood sample analysis;

  NOTE: ***Must note exact time of analysis (minutes and seconds preferred) using study's global time resource for reporting purposes. See Appendix IV for details.***
- Perform the second FMISO PET scan per the imaging parameters described in this protocol (see Section 10.0 and www.acrin.org/6684_imagingmaterials.aspx for imaging specifics on FMISO preparation, administration, and imaging parameters).
- Query participants for potential AEs. When an AE is reported, collect information about concomitant medication taken in the 2 weeks prior to the event and/or during the time of any reported AE. AE queries should be conducted by appropriate study-staff designee at the following time points:
  - At the time of injection;
  - Before leaving the PET suite;
  - If they call the site as instructed for any concerns during the 24 hours after FMISO administration;
  - By telephone after 24 hours post-FMISO infusion.

9.3 FOLLOW UP: Routine Disease Progression/Vital Status Assessment—Site Contacts Treating Physician Every 3 Months for Standard Clinical Follow-Up Findings and MRI/CT Images

Disease progression, vital status data, follow-up MRI and CT images, and image findings will be collected from all enrolled participants' treating physicians until the end of the study.

9.3.1 Participant clinical follow up will be conducted per institutional standard of care and per the recommendations of the participant's treating physician.

- Vital status, disease progression, and other data (e.g., tumor growth, neurological status, steroid use, steroid dose at the time of each MRI [or CT], treatment details, and interval treatments) will be collected every 3 months until the end of the study by the site from the treating physician's routine clinical follow-up findings.

  NOTE: Disease progression should be defined by Macdonald criteria[38] (see Appendix V for Macdonald Radiographic Response Criteria). Follow up will continue until the end of the study.

9.3.2 All standard-of-care MRIs and CTs for the participant taken during the trial timeframe will be requested every 3-months until the end of the study from the treating physician in addition to collecting clinical care follow-up information as described above in Section 9.3.1. Images will be submitted to ACRIN per the criteria described in Section 10.4 (Image Submission Criteria).

9.3.3 The end of the study will necessitate a final follow-up form for all surviving participants to provide confirmation of vital status and/or disease progression as of the last day/end of the study.

9.4 OFF-IMAGING CRITERIA

As long as at least the first baseline FMISO PET and MRI scans have been completed, participants will continue to be followed for disease progression and survival as outlined in Section 9.3.2. No participants will be considered "off-imaging" as long as the FMISO PET and MRI scans are completed prior to treatment initiation.

The trial will continue to accrue until evaluable images are available from 50 eligible participants.

9.5 STUDY PROCEDURES TABLE:

STUDY PROCEDURE	VISIT 1: Eligibility/ Registration or Screening Log	VISIT 2*: Baseline—Within 2 Weeks Prior to the Initiation of Chemoradiation	VISIT 2A†: 15 PARTICIPANTS ONLY—1 to 7 Days After Visit 2, Prior to Initiation of Chemoradiation	FOLLOW-UP: Every 3 Months Until End of Study
Informed Consent Form	X			
Eligibility/Registration	X			
Report Concomitant Therapy, If Scheduled (See NOTE in Section 9.1)	X			
Medical History	X			X
Physical Examination	X			
Assign Karnofsky and MMSE Mental Performance Scores	X	X		
Neurologic Examination	X			
Review Pathology Report, Operative Report, and Postop MR Image	X			
Screening Lab Work, Including Creatinine Levels for GFR±	X	X		
Vital Signs (Including Before FMISO Administration and After PET Scan)	X	X	X	
Urine Pregnancy Test	X	X	X	
Obtain & Send Tumor Tissue Specimen (If Feasible; See Section 18.0 for Details)	X			
Place 2 IV Catheters (1 in Each Arm)		X	X	
QC Well Counter		X	X	
Blood Sampling (3 Samples at 5-Minute [± 2 Minutes] Intervals Starting at Emission Scan Initiation)		X	X	
Note Exact Time of Blood Sample Collections and Sample Analyses		X	X	
Brain MRI With Contrast		X		X‡ (submit all follow up MRI and CT Scans)
FMISO PET (Saline Flush After FMISO Injection)		X	X†	
AEs Assessment With Telephone Follow-Up After 24 Hours Post-Infusion		X§	X§	

* **NOTE:** FMISO PET and MRI scans do not have to be completed on the same day; however, participants should be aware that 1 additional IV catheter will be needed for each additional day of scanning. Details are described in Section 9.2 above.

± To be completed if the creatinine levels were not completed within 28 days of registration and within 28 days prior to Visit 2 for the trial-related MRI scan with Gd contrast.

† Only 15 participants will undergo repeat FMISO PET scans, within 1 to 7 days of each other (independent of timing of baseline MRI scan) to evaluate reproducibility of FMISO PET.

‡ All MRIs (with or without contrast) and CT scans performed as standard of care during participant follow up should be submitted to ACRIN per the instructions in Section 10.4 (Image Submission Criteria).

§ A phone call will be made to participants after 24 hours after the FMISO injection to elicit AEs using open-ended questions. In the event an AE is reported, concomitant medication details from the 2 weeks prior to the event and/or during the time of the reported AE will need to be collected.

9.6 TREATMENT AND IMAGING SEQUENCE TABLE:

TREATMENT AND IMAGING		
	**Visit 2***	**Chemoradiation Begins**
	Baseline	Wk 1
Temozolomide (mg/m^2/day)		75
Radiation Therapy		•
FMISO PET Scan	•†	
Brain MRI Scan With Contrast‡	•	

* **NOTE:** FMISO PET and MRI scans do not have to be completed on the same day; however, participants should be aware that 1 additional IV catheter will be needed for each additional day of scanning. Details are described in Section 9.2 above.

† Only 15 participants will have 2 baseline FMISO PET scans performed 1 to 7 days apart for test-retest analysis (see Section 9.2.1 for details of Visit 2A); both scans must be completed prior to initiation of chemoradiation.

‡ All MRIs (with or without contrast) and CTs performed as standard of care during participant follow up should be submitted to ACRIN per the instructions in Section 10.4 (Image Submission Criteria).

10.0 IMAGING GUIDELINES

10.1 MR Imaging With Gadolinium Contrast

In order to participate in the FMISO trial, sites must be able to perform all of the advanced MRI series per vendor-specific parameters. Participating sites also must be able to capture and submit processed and raw spectroscopy file data. To ensure that the institution's scanners are able to perform the advanced imaging MRI/MRS sequences, all institutions must submit pre-qualification test scans to ACRIN using the scanner that will be used consistently throughout the trial. These test scans must be protocol compliant in order to become qualified to participate in the trial.

NOTE: All MRI scans must be completed at 1.5 Tesla or 3.0 Tesla; 3.0 Tesla is preferred. Participants **MUST** be scanned using the same ACRIN-approved MR scanner(s) used for trial qualification, using the same protocol-specific parameters consistently.

Detailed, vendor-specific technical parameters and site approval instructions are available via the ACRIN web site at www.acrin.org/6684_protocol.aspx.

10.1.1 MRI Sequences

Participating sites are required to complete all of the following MRI sequences for each participant for this FMISO trial (exact details available at the web site above):

- Scout;
- T1–weighted SE (pre injection);
- 3D T2 Rare;
- FLAIR;
- **OPTIONAL:** BOLD MRI during transient inhalation of oxygen in the magnet per institutional standard of care (minimum requirement 7 L/min) using nasal canula; 0_2 saturation levels will be collected during this sequence and documented;
- Dynamic contrast enhanced images with associated T1 mapping;
- Diffusion-weighted imaging;
- Dynamic susceptibility contrast imaging;
- Post T1 3D–weighted (1 mm isotropic);
- Post T1–weighted SE;
- 3D volumetric spectroscopy is preferred. 2D CSI spectroscopy is accepted. Additional details are available online.

10.1.2 MRI Contrast Agent, Gadolinium

Gadolinium contrast will be administered according to current clinical standard-of-care practice and using only FDA-approved agents. In reflection of recent FDA label changes for several gadolinium agents, trial leadership advises caution in using gadolinium contrast agents in patients with poor kidney function and encourages education for participants in the signs and symptoms of nephrogenic systemic fibrosis (NSF). Sites should feel free to contact trial leadership if there are any questions either about NSF in general or about the eligibility of a given patient with respect to this issue.

Each IRB must approve this study, including its gadolinium contrast dosage. Allowed contrast agents will be gadopentate dimeglumine (Magnevist), gadoteridol (ProHance), gadodiamide (OmniScan), gadoterate meglumine or gadoversetamide (Optimark),

depending on each site's clinical practice. We seek to respect individual site's decisions on the issue of choice of contrast agent, an issue that has garnered substantial discussion in the past three years due to the recognition of associated risks of nephrogenic systemic fibrosis. The contrast agent will be administered at a dose of 0.1 mmol/kg (for dynamic contrast enhanced MRI) followed by 0.1 mmol/kg (for dynamic susceptibility contrast MRI) injected at 3 to 5cc/sec as is current clinical practice and only upon approval by the IRBs in this trial. We note that one of the agents (ProHance) is explicitly for a dose of up to 0.3 mmol/kg ("triple dose"); use of other agents will be under local IRB approval. Gadobenate dimeglumine (MultiHance) and gadofosveset (marketed as Vasovist in Europe) are not preferred in this trial because their binding to serum albumin may influence DCE MRI measurements in unknown ways.

10.1.3 MRI Quality Assurance Review

- All institutions must complete qualification procedures to establish an ACRIN-approved MRI scanner prior to registering participants.
- Qualification requires sites to submit for review one MRI and one MRS examination, performed per protocol specification using the parameters available on the ACRIN web site at www.acrin.org/6684_protocol.aspx. All submissions must include raw spectroscopy files (e.g. GE: P-files; Siemens: .rda files; Philips: .spar/.sdat files). Raw MRS data files can be transmitted to the ACRIN Core Laboratory via secure File Transfer Protocol (sFTP) directly to the image archive. All MRS submissions must also include an image demonstrating voxel placement. Vendor-specific instructions for locating and submitting raw MRS data can be found on the ACRIN web site under Imaging Requirements at www.acrin.org/6684_protocol.aspx.

10.2 FMISO PET SCAN

Participant must be scanned on PET scanners that have been qualified by the ACRIN PET Core Laboratory per the protocol-specific instructions posted on the ACRIN web site at: www.acrin.org/CORELABS/PETCORELABORATORY/PETQUALIFICATION/tabid/485/Default.aspx.

A dedicated PET scan unit or hybrid PET/CT scanner is mandatory. The PET scanner must be capable of performing both emission and transmission images in order to allow for attenuation-corrected PET scan images. The ability to calculate standardized uptake values (SUVs) is also mandatory.

Serial scans of the same participant must be done on the same scanner for this study.

The PET scanner must be kept calibrated in accordance with the manufacturer's recommendations. The scanner should routinely be assessed for quantitative integrity and stability by being tested using various imaging protocols on a standard phantom. For SUV measurements, this assessment should include a comparison against a dose calibrator to ensure accuracy; that is, a comparison of the absolute activity measured versus the measured activity injected, should be performed.

The PET scanner calibrations should be routinely verified according to manufacturer recommendations. The scanner should be assessed regularly for quantitative integrity and

stability by scanning a standard quality control phantom with the same acquisition and reconstruction protocols used for study participants. The SUV verification measurements must include the dose calibrator used to measure the doses of study participants to ensure that the dose calibrator and PET scanner are properly cross calibrated, i.e. the dose measured in the dose calibrator and injected into the phantom matches the results obtained from analysis of the phantom images.

A quality control (QC) check must be performed at the beginning of the day for the dose calibrator and well counter, in accordance with the manufacturer recommendations. If any of the QC results are outside of the manufacturer's guidelines, the study must be rescheduled and the problem rectified before scanning any patients.

Exact time collection is key to this protocol. A global time piece should be used consistently as a standardized measuring tool—such as a portable stop watch—throughout the trial for all time collection. The time piece will need to accurately record minutes, at the least, and preferably seconds as well. It will be used to collect time at dose counting, injection time, imaging start time, blood sampling time, and blood counting time.

10.2.1 Pre-Scan Participant Preparation

- There will be no deliberate fasting prior to injection for the participant of this study.
- Participants are encouraged to be well hydrated prior to the scan.
- Blood glucose measurement is not required.
- Participant's height and weight must be measured using calibrated and medically approved devices (not verbally relayed by the participant).
- Vital signs, including temperature, blood pressure, heart rate, and respiratory rate, will be measured prior to injection.

10.2.2 Injection of FMISO

- Two IV catheter access lines (18 or 20 gauge is preferred) are placed, one in each arm—one for the FMISO injection and the other for blood sampling during the scan.
- The dose of FMISO will be 3.7 MBq/kg (0.1 mCi/kg) (maximum 260 MBq, 7 mCi) in $\leq$ 15 mL. For the FMISO injection, minimize the length of the IV tubing between the injection site and the vein to avoid FMISO being left in the tube.
- A saline flush should follow the FMISO injection.
- The exact time of calibration of the dose should be recorded using a global time piece consistently used throughout the study for time recording; the exact time of injection should be noted and recorded to permit correction of the administered dose for radioactive decay. In addition, any of the dose remaining in the tubing or syringe, or that was spilled during injection, should be recorded. The injection should be performed through an IV catheter and 3-way stopcock.
- AEs will be solicited in open-ended fashion (i.e., "how are you feeling?") at this time.

10.2.3 FMISO PET Imaging

- Single field-of-view emission imaging must begin 110 ± 10 minutes after FMISO injection. Imaging start time should be recorded using the global time piece for the study.

- The participant will empty his/her bladder immediately before the acquisition of images.
- Participants are imaged in the supine position with head immobilization. Head immobilization procedures for PET imaging will be site-specific.
- An attenuation scan should be performed prior to emission scanning, or just after emission scanning if there is a problem before. The transmission scan should be a low-dose CT scan without contrast for the PET/CT or a transmission scan for PET-only devices. The transmission scan type, length, etc, should exactly match that used in the calibration and qualification procedure.
- A 20-minute emission scan is performed.
- During emission tomography, three (3) venous blood samples are obtained at intervals of 5 minutes (± 2 minutes). Blood must be drawn from the IV that was not used for the FMISO injection. The exact time of the blood draws should be recorded using the standard global time piece for the study.
- Whole blood samples of 1 mL each are counted (within 60 minutes of collection) in a multichannel gamma well counter that is calibrated quarterly, or as specified by the manufacturer, in units of cpm/uCi (see Appendix III for well counter cross-calibration instructions and Appendix IV for blood sampling procedures).
- The exact time that each blood sample is counted should be recorded using the global time piece.
- Blood activity is averaged and then expressed as uCi/mL decay corrected to time of injection.
- Vitals signs, including temperature, blood pressure, heart rate, and respiratory rate, are measured again at completion of the FMISO PET imaging, and subsequently as needed.

Figure 1. Timing Sequence for FMISO Injection and PET Imaging

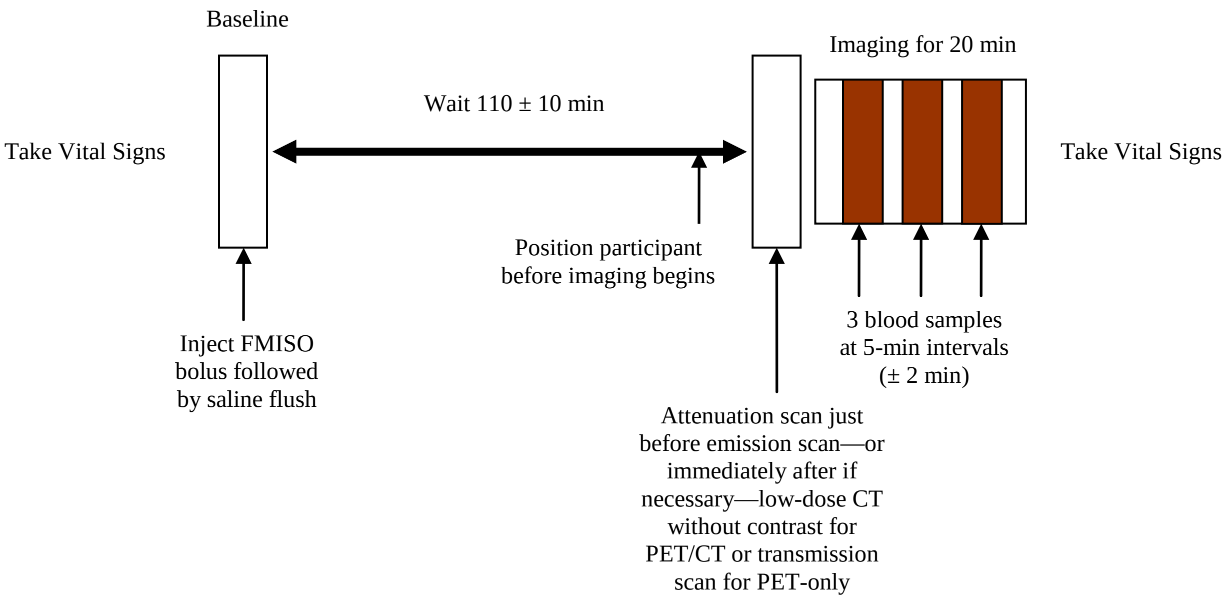

NOTE: Global time will need to be recorded for dose counting, injection time, imaging start time, blood sampling time, and blood counting time throughout the trial.

10.2.4 Image Reconstruction

- The PET data will be corrected for dead time, scatter, random, and attenuation using standard algorithms provided by the scanner manufacturer.
- Image reconstruction will be performed as specified in the ACRIN certification of the PET scanner.

10.3 Image Analysis

MRIs are co-registered with the FMISO images to delineate tumor and brain regions of interest (ROI). The FMISO image data and blood sample are expressed in common units of uCi/mL. Image values are divided by the average blood value to produce tissue/blood (T/B) values. This allows a pixel-by-pixel calculation of T/B activity ratios for all image planes. The number of pixels in the tumor volume with a T/B ratio $\geq$ 1.2, indicating hypoxia, is multiplied by the known volume/voxel for the scanner to yield milliliter units to measure the HV, based upon prior studies. In the tumor region we also locate the pixel with the maximum T/B ratio and record this variable, T/B_{max}. HV depicts the volume of tumor that has crossed the threshold for hypoxia and T/B_{max} depicts the magnitude of the hypoxia.

10.4 Image Submission Protocol

The protocol-required images must be in DICOM format on CD/DVD-ROM or submitted via the internet using sFTP, and transmitted along with an Imaging Transmittal Worksheet (ITW) which can be found on the ACRIN web site (www.acrin.org/6684_protocol.aspx). The required images

must be submitted to the ACRIN core lab. ACRIN can provide electronic image submission and anonymity utilities for participating institutions via TRIAD software. For support in sending the images via the internet using TRIAD, contact the representatives of the core lab via email at Triad-Support@phila.acr.org or via phone: 215-940-8820.

10.4.1 If required and part of the protocol, images maintained at ACRIN Headquarters Image Archive may be distributed to other participating sites, using sFTP, or CD-ROM where appropriate, for purposes of secondary review.

10.4.1.1 Removal of Confidential Participant Information: The header record on DICOM formatted image data, which often contains information identifying the participant by name, MUST be scrubbed before the image are transferred.

This involves **replacing** the following:

- Participant Name tag with the ACRIN Institution ID or number,
- Participant ID tag with the ACRIN case number, and
- Other Participant ID tag with ACRIN Study Number.

10.4.1.2 sFTP Transfer: Digitally generated image files in DICOM v3.0 format can be transmitted to the ACRIN core lab via sFTP directly to the image archive. This can be performed using a customized software program or by using TRIAD software available from ACRIN. An Imaging Transmittal Worksheet (ITW) must be faxed at the time images are transmitted. Contact the ACRIN core lab for additional details at Triad-Support@phila.acr.org.

10.4.1.3 A completed, signed ITW MUST accompany all imaging exams submitted to ACRIN for each time point. The ITW must be completed and faxed to 215-923-1737 at the time the images are being submitted. For exams submitted via media, this worksheet may be completed and included with the media shipment. Please affix a label to the jacket of the media to include: study name, site name, and case number, date of exam, time point, and type of imaging.

Please fax the ITW to:

ACRIN Core Lab at 215-923-1737
ATTN: ACRIN 6684 Imaging Specialist

10.4.1.4 In the event that the transfer of scrubbed image headers is not available, images may also be sent on a CD/DVD-ROM to the ACRIN core lab for transfer to the image archive. Please contact ACRIN prior to sending the media to confirm compatibility.

Images and the ITW may be mailed to:

American College of Radiology Imaging Network
Core Laboratory
Attn: ACRIN 6684
1818 Market Street 16th floor
Philadelphia, PA 19103

10.5 Spectroscopy Data Submission

Raw MRS data files can be transmitted to the ACRIN core lab via sFTP directly to the image archive. All MRS submissions must include an image demonstrating voxel placement. Vendor-specific instructions for locating and submitting raw MRS data can be found on the ACRIN web site under Imaging Materials at www.acrin.org/6684_imagingmaterials.aspx.

10.6 Image Quality Control

All image submissions will be subject to ongoing quality control review by the ACRIN core lab. In addition, a central quality review of the first two imaging submissions from each participating institution will be formally conducted. Thereafter, a random sampling of 10% of the remaining cases will be formally reviewed.

Prompt submissions of all image data are essential to ensure adequate image quality control.

11.0 WELL-COUNTER CALIBRATION/BLOOD SAMPLING GUIDELINES

Exact time of collection is key for this protocol. Time will need to be recorded from a global resource to be used throughout the study. Preferably, minutes and seconds will be recorded and reported.

11.1 Well-Counter Calibration

See www.acrin.org/6684_imagingmaterials.aspx for additional procedural details.

11.1.1 As a pre-qualification measure, sites will need to demonstrate appropriate well-counter cross calibration.

11.1.2 Well-counter cross calibration should be completed quarterly or as specified by the manufacturer. (Routine well-counter QC measures will still be necessary on the day of imaging/counting.)

11.1.3 An Excel document to assist in calculations during cross calibration is provided online at www.acrin.org/6684_imagingmaterials.aspx.

11.2 Blood Sampling

See Appendix IV for additional procedural details.

11.2.1 Blood will be drawn from all participants during the 20-minute FMISO PET imaging sequence. Figure 1 presents the timeline for the FMISO PET imaging sequence, including blood sampling every 5 minutes (± 2 minutes) at 3 time points (e.g., at minutes 5, 10, and 15 after FMISO emission scan begins).

11.2.2 Sites are expected to process the blood samples within 60 minutes after completion of the PET scan.

11.2.3 Use of dose calibrators for this procedure is strictly prohibited.

12.0 ADVERSE EVENTS REPORTING

Adverse Event (AE) reporting must follow the guidelines below. The ACRIN Adverse Event Reporting Manual [November 2009 version or latest revision—available on the ACRIN web site] provides additional details and may be consulted as a reference, but does not supersede AE reporting as specified in this protocol. The AdEERS electronic AE reporting system is to be used for AE reporting of AdEERS qualified events unless unavailable. AE data are to be entered into the AdEERS electronic system if the AE meets AdEERS criteria (see Section 12.8 and 12.9 for AdEERS reporting criteria), even when they have been manually captured and/or manually submitted due to system unavailability (see manual process below).

12.1 Definition of Adverse Event

An **Adverse Event (AE)** is any untoward medical occurrence in a participant that does not necessarily have a causal relationship with the study intervention. An AE can therefore be any unfavorable and unintended sign (including an abnormal laboratory or physiological finding), symptom, or disease temporally associated with the use of a medical treatment or procedure, regardless of whether it is considered related to the medical treatment or procedure (attribution of unrelated, unlikely, possible, probable, or definite). Abnormal results of diagnostic procedures are considered to be AEs if the abnormality:

- Results in study withdrawal;
- Is associated with a serious adverse event (SAE);
- Is associated with clinical signs or symptoms;
- Leads to additional treatment or to further diagnostic tests;
- Is considered by the investigator to be of clinical significance.

12.2 Definition of Serious Adverse Event

A Serious Adverse Event (SAE) is defined as any untoward medical occurrence that meets any one of the following criteria:

- Results in death or is life-threatening at the time of the event; <u>or</u>
- Requires inpatient hospitalization, or prolongs a hospitalization; <u>or</u>
- Results in a persistent or significant disability/incapacity; <u>or</u>
- Is a congenital anomaly/birth defect (in a participant's offspring); <u>or</u>
- Requires intervention to prevent any of the above, per the investigator/sponsor.

A **life-threatening AE** is an AE that places the study participant, in the clinical opinion of the investigator, at immediate risk of death.

12.3 Adverse Event Grading

Grade denotes the severity of the AE. An AE is graded using the current version of the Common Terminology Criteria for Adverse Events (CTCAE) version 4.0, or the following categories (if the term does NOT appear in the CTCAE v4.0):

1 – Mild
2 – Moderate
3 – Severe

4 – Life-threatening or disabling
5 – Fatal

(For terms listed in the CTCAE, the grade is still recorded as 1, 2, 3, 4, or 5; however, the definition of the various grades will be specific to the term being used.)

12.4 Adverse Event Attribution

Attribution determines whether an AE is related to a study treatment or procedure. Attribution categories are:

Definite – AE ***is clearly related*** to the study treatment or procedure.
Probable – AE ***is likely related*** to the study treatment or procedure.
Possible – AE ***may be related*** to the study treatment or procedure.
Unlikely – AE ***is doubtfully related*** to the study treatment or procedure.
Unrelated – AE ***is clearly NOT related*** to the study treatment or procedure.

12.5 Expected and Unexpected Adverse Events

AEs may be **expected** or **unexpected**:

- An **expected AE** is one that is described in the protocol, the ICF, or the investigator's clinical brochure, even if such an event would be considered extremely rare.
- An **unexpected AE** is one that has not been described in the protocol, the ICF, or the investigator's clinical brochure.

12.6 Expected Adverse Events From MRI Scans With Contrast and FMISO PET Scans

12.6.1 Expected Adverse Events Associated With MRI

- Anxiety/stress;
- Claustrophobia;
- Discomfort.

Because of the powerful magnetic force of the MRI scanner, the patient may be ineligible to participate in the study if he or she has:

- Metallic or other surgical implants (for example: pacemaker, heart valves, aneurysm clips, metal plates or pins and some orthopedic prostheses);
- Metal pieces in your eye(s) or other body part(s); or
- Difficulty lying still or inability to lie on your stomach.

12.6.2 Expected Adverse Events From the Gadolinium Contrast Agent (Gd):

Risks Related to Renal Dysfunction and Hemolytic Anemia

Precautions should be exercised for participants with severely impaired renal function (see below) or hemolytic anemia. The very unlikely possibility of a reaction, including anaphylactic or cardiovascular reactions, should be considered especially for participants with a known sensitivity to Gd or history of asthma.

Nephrogenic Systemic Fibrosis (NSF) or Nephrogenic Fibrosing Dermopathy (NFD), kidney disorders, may occur in participants with moderate to end-stage kidney disease (glomerular filtration rate <30mL/min/1.73m^2) and in participants with renal dysfunction due to the hepatorenal syndrome or in the perioperative liver transplantation period after they have had a MRI scan with Gd-based MR contrast agents (GBMCA).

NSF causes fibrosis of the skin and connective tissues throughout the body. Participants develop skin thickening that may prevent bending and extending joints, resulting in decreased mobility of joints. NSF usually starts in the lower extremities. Fibrosis can also develop in the diaphragm, muscles in the thigh and lower abdomen, and lung vessels. Reference: FDA/Center for Drug Evaluation and Research. May 23, 2007 (www.fda.gov/cder/drug/infopage/gcca/qa_200705.htm).

Other Gd-Related Risks:

- Nausea;
- Headache;
- Hives;
- Temporary low blood pressure;
- Allergic reaction.

12.6.3 Expected Adverse Events From IV Needle Placement:

- Hemorrhage (hematoma at the injection site);
- Infection (catheter related infection) at the injection site;
- Minor discomfort;
- Bleeding;
- Bruising.

12.6.4 Expected Adverse Events Associated With Radiation Risks:

The radiation dosage for PET scanning varies with the part of the body being scanned, the source of the attenuation scan, the timing of the scan, the type of PET imaging being performed, and institution-specific radiation safety policies. The range of exposure for PET scanner can therefore be wide.

For ACRIN 6684, all PET exposures for the radiopharmaceutical [^{18}F]FMISO and all PET imaging will be performed for the head/brain only. Attenuation for PET correction can be performed using a calculated attenuation map (no radiation exposure), a radioisotope transmission source (PET only devices), or a CT scan (PET/CT devices). The following ranges have been estimated by calculating CT transmission doses using the ImPACT program from the Medicines Device Agency in the United Kingdom and represent the best estimates available to trial leadership at this time for FMISO used at a maximum does of 7 mCi.

For participants who undergo two FMISO PET scans (15 participants who undergo the test-retest scans at baseline), the exposure for the FMISO PET examinations in this study are likely to range from a minimum of 7.8 mSv (780 mrem) to a maximum of 9.8 mSv (980 mrem) (see Table 3 below). For all other participants who have only one FMISO

PET scans, the likely dose is a minimum of 3.9 mSv (390 mrem) to a maximum of 4.9 mSv (490 mrem).

The radiation dose from the PET has not been shown to have any adverse effects. Table 3 below describes the radiation exposure associated with each individual scan for this trial based on data collected in a survey of current attenuation correction practices and effective radiation doses conducted by the NCI Cancer Imaging Program in conjunction with several leading ACRIN research centers, as reported in the report, *Estimation of Radiation Doses Due to PET Radiopharmaceuticals and to Attenuation Correction of PET Images*, and applied to the FMISO PET scans planned for the ACRIN 6684 trial.

For each PET scan included in ACRIN 6684, radiation dose estimates are provided for the maximum possible administered FMISO dose (7 mCi) and one of four scenarios:

(1) Typical CT-based attenuation scan;

(2) Maximum dose from CT-based attenuation scan from the institutional survey;

(3) PET-only device transmission scan; and

(4) Calculated attenuation scan (no transmission scan exposure).

These approaches are representative of the range of technique used at ACRIN 6684 participating sites.

Table 3. Radiation Exposure in ACRIN 6684

FMISO PET Scan Visit(s)	Eligible Patients	FMISO PET Agent Dose	FMISO ED for Maximum Dose (mSv)	Equipment Type	FMISO PET/CT Image Acquisition (head)	Attenuation Transmission Scan	ED from CT scan (mSv) (Mean)	ED from CT scan (mSv) (Max)	ED from □□Trans-mission Scan (mSv) (Max)	Total ED (mSv) (Likely)	Total ED (mSv) (Max)	ED with Trans-mission Scan Only (mSv)	ED with No Trans-mission (PET/MRI)
**Prior to Start of Chemoradiation (Baseline)**	All	3.7 MBq/kg (0.1 mCi/kg) maximum 260 MBq (7 mCi)	3.9	PET/CT or PET	Single field-of-view emission imaging at 110 ± 10 minutes post injection	Low-dose CT scan or transmission	0.6	1.0	0.02	4.5	4.9	3.9	3.9
**2nd FMISO PET Baseline Scan (Within 1–7 Days Reproducibility Test)**	Subset: 15	3.7 MBq/kg (0.1 mCi/kg) maximum 260 MBq (7 mCi)	3.9	PET/CT or PET	Single field-of-view emission imaging at 110 ± 10 minutes post injection	Low-dose CT scan or transmission	0.6	1.0	0.02	4.5	4.9	3.9	3.9
**2 Scan**	15	≤ 14 mCi	7.8				1.2	2.0	0.04	9.0	9.8	7.8	7.8
**1 Scan**	**All**	**≤ 7 mCi**	3.9				0.6	1.0	0.02	4.5	4.9	3.9	3.9

ED = Estimated Effective Dose
Likely dose = radionuclide + mean from CT scan (see report *Estimation of Radiation Doses Due To PET Radiopharmaceuticals and to Attenuation Correction of PET Images*)

12.6.5 Expected Adverse Events From PET Scan:

- Discomfort;
- Claustrophobia.

12.6.6 Expected Adverse Events Associated With FMISO Injection:

(NSC #742836; IND #76,042)

Comprehensive Adverse Events and Potential Risks List (CAEPR)
for
[^{18}F]Fluoromisonidazole, (FMISO, NSC 742836)

The Comprehensive Adverse Event and Potential Risks list (CAEPR) provides a single list of reported and/or potential adverse events (AE) associated with an agent using a uniform presentation of events by body system. In addition to the comprehensive list, a subset, the Agent Specific Adverse Event List (ASAEL), appears in a separate column and is identified with ***bold*** and ***italicized*** text. This subset of AEs (ASAEL) contains events that are considered 'expected' for expedited reporting purposes only. Refer to the 'CTEP, NCI Guidelines: Adverse Event Reporting Requirements' http://ctep.info.nih.gov/protocolDevelopment/default.htm#adverse_events_adeers for further clarification. The CAEPR does not provide frequency data; refer to the Investigator's Brochure for this information. Below is the CAEPR for [^{18}F]Fluoromisonidazole.

Version 1.0, July 1, 2010 [1]

**Category (Body System)**	**Adverse Events[2] with Possible Relationship to [^{18}F]Fluoromisonidazole (CTCAE v4.0 Term)**	**EXPECTED AEs FOR ADEERS REPORTING Agent Specific Adverse Event List (ASAEL)**
	No AEs reported in human studies.	

[1]This table will be updated as the toxicity profile of the agent is revised. Updates will be distributed to all Principal Investigators at the time of revision. The current version can be obtained by contacting PIO@CTEP.NCI.NIH.GOV. Your name, the name of the investigator, the protocol, and the agent should be included in the e-mail.

Note: No adverse events have been attributed to Positron-Emission Tomography (PET) imaging/diagnostic administration of [^{18}F]Fluoromisonidazole at the levels described in the Investigators Brochure. Therefore, no adverse events are expected as a result of the intravenous (IV) administration of [^{18}F]Fluoromisonidazole for typical PET imaging applications such as tumor hypoxia.

Note: As with many IV administered agents, [^{18}F]Fluoromisonidazole could cause an allergic reaction that could potentially pose a threat to life (anaphylaxis). This has not been observed in limited human exposure to date. Reasonable precautions should be taken, consistent with normal radiologic and clinical facility practice. The patient should be monitored until the PET

procedure is completed, and trained personnel and emergency equipment should be available per facility standards.

For purposes of informed consent regarding reasonably foreseeable risks to subjects in trials utilizing [^{18}F]Fluoromisonidazole, the following potential adverse events are considered extremely rare:

- **Injection-related risks that may include infection, or accidental extravasation of the dose that may lead to discomfort, localized pain, or infection.**
- **Risks related to allergic reaction/anaphylaxis that may be life threatening.**

Note: As with all PET imaging agents, [^{18}F]Fluoromisonidazole is a radiopharmaceutical that decays with positron emission. As such, it poses an intrinsic radiation exposure risk. However, when administered in accordance with the Investigator's Brochure as a PET imaging agent, this risk is felt to be extremely small. The organ and total body doses associated with [^{18}F]Fluoromisonidazole PET imaging are comparable to or lower than those associated with other widely used clinical nuclear medicine procedures.

Note: [^{18}F]Fluoromisonidazole in combination with other agents could cause an exacerbation of any adverse event currently known to be caused by the other agent, or the combination may result in events never previously associated with either agent.

12.6.7 Adverse Events Related to Chemotherapy and Radiation Therapy:

ACRIN will not collect any AEs related to chemotherapy and radiation therapy. Sites will follow institutional policies and procedures for any reported side effects for chemoradiation therapy.

ACRIN will collect AEs that occur after 24 hours of FMISO infusion regardless of attribution/relatedness. Beyond this period, AEs that are determined to be definitely, probably, or possibly related to the participants' underlying disease or therapy, that are ALSO determined to be unrelated or unlikely related to the FMISO and procedures specified in this protocol, will not be reported. Therefore, most AEs related to chemoradiation treatment are not expected to be reported for this trial.

12.7 Reporting of Adverse Events

The descriptions and grading scales found in the revised NCI CTCAE v4.0 will be utilized for AE reporting. All institutions should have access to a copy of the CTCAE v4.0. A copy of the CTCAE v4.0 can be downloaded from the CTEP web site: http://ctep.cancer.gov/reporting/ctc.html. A list of AEs that have occurred or might occur (Reported Adverse Events and Potential Risks) can be found in Section 6 (Pharmaceutical Information).

Prompt reporting of AEs is the responsibility of each investigator, clinical research associate (RA), and/or nurse engaged in clinical research. Anyone uncertain about whether a particular AE should be reported should contact the ACRIN headquarters at 215-574-3150 for assistance. This study will be monitored by ACRIN and by the NCI/CIP.

All AEs should be followed by the investigator until the events are resolved, the subject is lost to follow-up, the condition is stabilized, or the AEs are otherwise explained. Any death or any AE

(e.g. development of cancer, congenital anomaly in conceived offspring) that is determined to be possibly, probably, or definitely related to study agents or procedures as specified in this protocol—even if it occurs after a subject has discontinued study participation—must be reported to the ACRIN AE Coordinator at DMC and to all appropriate study monitors and applicable regulatory authorities as specified herein or as required by regulation or guidance.

Assignment of grades and attribution for each AE/SAE must be completed by the site principal investigator or investigator-designee. All AEs/SAEs must be documented in the participant's study chart, AE form, and events that qualify for AdEERS must be submitted through the electronic-AdEERS (e-AdEERS) system in the required timeframes, as specified below. The ACRIN RA will capture all AEs as required herein on the AE form and in the e-AdEERS system where appropriate. This will ensure that they are provided to the NCI/CIP Monitor and will also ensure their receipt by the AE Coordinator at the DMC. Expedited AdEERS reports must be entered by the investigator or investigator-designee. The expedited AdEERS report will be submitted to the lead group reviewer at ACRIN and then submitted once reviewed to the NCI/CIP. A copy of the report must be kept on file at the site. Significant new information on any on-going AE/SAE should be promptly reported to NCI/CIP and ACRIN.

At each contact (site visit and/or telephone) with the study participant, the investigator or investigator-designee must elicit, through open ended questioning (e.g. "How are you feeling?") information on AEs and, if indicated, the subject should be evaluated clinically. When AEs are reported, concomitant medication taken by the participant prior to 2 weeks and/or during the time of the AE will be collected and documented. Information on all AEs that occur after 24 hours of FMISO infusion should be recorded immediately into the source document (e.g. ACRIN AE Log and/or progress notes of the participant's study chart) and retained at the site. These AEs will also be recorded in the AE form. SAEs and AEs requiring expedited reporting will be entered into the e-AdEERS system as required, pending review by the principal site investigator in real time to determine grade and attribution of the event and timeframe for submission to ACRIN.

All SAEs that are still ongoing at the end of the study must be followed up to determine, to the degree possible, the final outcome. Any SAE that occurs after the study period and is considered to be possibly related to the study procedures or study participation should be recorded and reported immediately.

12.8 E-AdEERS Expedited Reporting Requirements for Adverse Events That Occur After 24 Hours of a Dose of the Investigational Agent: FMISO

Table 4. Reporting Requirements for Adverse Events That Occur Within 24 Hours (± 4 Hours) of a Dose of the Investigational Agent (FMISO)[1]

Attribution	**Grade 1**	**Grade 2**	**Grade 2**	**Grade 3**		**Grade 3**		**Grades 4 & 5[2]**	**Grades 4 & 5[2]**
	**Unexpected and Expected**	**Unexpected**	**Expected**	**Unexpected**		**Expected**		**Unexpected**	**Expected**
				**with Hospitali-zation**	**without Hospitali-zation**	**with Hospitali-zation**	**without Hospitali-zation**		
**Unrelated Unlikely**	Not Required	Not Required	Not Required	10 Calendar Days	Not Required	10 Calendar Days	Not Required	10 Calendar Days	10 Calendar Days
**Possible Probable Definite**	Not Required	10 Calendar Days	Not Required	10 Calendar Days	10 Calendar Days	10 Calendar Days	Not Required	24-Hour; 5 Calendar Days	10 Calendar Days

[1] In addition to the above, **Grade 4 and 5 unexpected adverse events with attribution of possible, probable, or definite** with regard to FMISO, that occur more than 24 hours after administration of FMISO require e-AdEERS 24-hour notification followed by complete report within 5 calendar days.

e-AdEERS 10 calendar day report:

- Grade 2 unexpected events with attribution of possible, probable, and definite
- Grade 3 unexpected events with hospitalization or prolongation of hospitalization with attribution of unrelated and unlikely and possible, probable, and definite
- Grade 3 unexpected events without hospitalization or prolongation of hospitalization with attribution of possible, probable, and definite
- Grade 3 expected events with hospitalization or prolongation of hospitalization with attribution of unrelated and unlikely and possible, probable, and definite
- Grade 4 and 5 unexpected events with attribution of unrelated and unlikely
- Grade 4 and 5 expected events with attribution of unrelated and unlikely and possible, probable, and definite

[2] Although an e-AdEERS 24-hour notification is not required for death clearly related to progressive disease, a full report is required as outlined in the table.

For **Hospitalization** only—Any medical event equivalent to CTCAE grade 3, 4, or 5 that precipitated hospitalization (or prolongation of existing hospitalization) must be reported regardless of designation as expected or unexpected and attribution.

March 2005

NOTE: All subjects' deaths that occur during the study require both routine and expedited reporting regardless of causality. Attribution to treatment or other cause should be provided.

12.8.1 Expedited AE Reporting Timelines

- **24 Hours; 5 calendar days** – The ACRIN investigator or investigator-designee must initially report the AE via e-AdEERS within 24 hours of first knowledge of the event followed by a complete e-AdEERS report within 5 calendar days of the initial 24-hour report.
- **10 calendar days** – A complete e-AdEERS report on the AE must be submitted within 10 calendar days of the ACRIN investigator or investigator-designee's first knowledge of the event.

  - Any medical event equivalent to CTCAE v4.0 grade 3, 4, or 5 that precipitates **hospitalization* (or prolongation of existing hospitalization)** must be reported regardless of attribution and designation as expected or unexpected with the exception of any events identified as protocol-specific expedited adverse event reporting exclusions.

- Any event with an IND imaging agent–related attribution of **possible, probable,** or **definite** that results in **persistent or significant disability/incapacity, congenital anomaly, or birth defect** must be reported via e-AdEERS by ACRIN if the event occurs following treatment with an agent under a CIP IND.
- Expedited reporting is defined as immediate notification to ACRIN and NCI/CIP through submission of an e-AdEERS report. Some AEs require 24-hour notification via e-AdEERS. When Internet connectivity is disrupted, a 24-hour notification is to be made to the CIP monitoring service, as specified below, by telephone. Routine reporting requirements also apply.[†]

* Hospitalizations are defined as lasting 24 hours or longer and these events must be reported via e-AdEERS.

† Routine reporting is defined as documentation of AEs on source documents and the AE CRF. Documentation should be submitted to ACRIN for preparation of a report for DMC review, quarterly reports to CDUS, FDA annual reports, and the final study report.

12.9 How to Report

NOTE: The e-AdEERS system routes AE reports to the necessary recipients/reviewers. The following contact information is provided to indicate the per-protocol recipients, forwarding timeframes, and distribution in the event this process must be done manually:

12.9.1 Some AEs require 24-hour notification (refer to Table 4 in Section 12.8) via e-AdEERS. When Internet connectivity is disrupted, a 24-hour notification is to be made as follows:

- Fax AE submission form and any additional information[1] necessary for thorough review of the event(s) to 301-897-7402, attention CIP SAE Team. The phone number for the CIP SAE Reporting Desk is 301-897-1704.
- Follow up with an e-mail to CIPSAEReporting@tech-res.com notifying that an AE form and additional information (if available) has been faxed.

Once Internet connectivity is restored, a 24-hour notification that was faxed or phoned in must be entered electronically into AdEERS by the original submitter at the site.

When the AE requires expedited reporting, submit the report within the number of calendar days of first knowledge of the event specified in Table 4 in Section 12.8. An expedited AE report requires submission to NCI by the DMC via the e-AdEERS system. CIP will then be notified via e-AdEERS and ACRIN of the expedited reporting. An expedited e-AdEERS report will be submitted to the lead group reviewer at ACRIN and then submitted once reviewed to the NCI/CIP.

[1] For events unrelated to the imaging intervention, no additional information is necessary. For events possibly, probably, or definitely related to the imaging intervention, additional information should include, admission and discharge summaries (when available), laboratory workup, progress notes, circumstances of the event(s).

12.9.2 Recipients of e-AdEERS Reports:

e-AdEERS Reports are electronically submitted to the following trial personnel; in the event that Internet connectivity is disrupted, AdEERS reports should be submitted as in Section 12.9.1 (above) and faxed to the attention of:

To ACRIN:
Maria Oh: moh@acr.org
Attention: ACRIN SAE Coordinator
RE: Adverse Event Report, ACRIN 6684
ACRIN Protocol 6684
1818 Market Street
Suite 1600
Philadelphia, PA 19103

CIP SAE Reporting Desk:
Phone: 301-897-1704
Fax : 301-897-7402
Email: CIPSAEReporting@tech-res.com
ATTN: CIP Support S&P Manager, Anna Edouard, MD

12.9.3 All fatal AEs identified in the FMISO PET scan component of the study must be reported by telephone within 24-hours of first knowledge of the event. To make a telephone report, call ACRIN at **215-717-2763**, available 24 hours a day (recorder after hours Monday through Friday from 4:30 PM to 8:30 AM Eastern Time and on weekends).

12.9.4 All fatal AEs identified in the FMISO PET scan component of the study must also be reported to CIP by telephone at **301-897-1704** within 24-hours of first knowledge of the event and via e-AdEERS.

12.9.5 All expedited AE reports should be sent to the local Institutional Review Board (IRB). Refer to the IRB policies and procedures for AE reporting.

12.9.6 For automated CDUS reporting for studies using investigational agents, the DMC routinely reports AEs electronically to the CTEP Clinical Data Update System (CDUS Version 3.0). The DMC submits this data quarterly. The AEs reported through AdEERS will also be included with the quarterly CDUS data submissions.

13.0 ETHICAL CONSIDERATIONS

This study is to be conducted according to US and international standards of Good Clinical Practice (International Conference of Harmonisation [ICH] guidelines), applicable government regulations, and ACRIN research policies and procedures.

This protocol and any amendments will be submitted to a properly constituted independent Ethics Committee (EC) or Institutional Review Board (IRB) for formal approval of the study conduct. The decision of the EC/IRB concerning the conduct of the study will be made in writing to the investigator and a copy of this decision will be provided to ACRIN before implementation of the study.

The investigator will provide ACRIN with the institution's federal wide assurance (FWA) number, along with the IRB approval letter and copy of the IRB-approved ICF. The investigator will provide a copy(s) of IRB approval letter(s) for any amendment(s), and copy(s) of annual renewal(s).

All study participants in this study will be given an IRB-approved, site-specific ICF describing the study and providing sufficient information for participants to make informed decisions about their participation in this study (see Appendix I for an ICF template). The ICF will be submitted along with the protocol for review and approval by the EC/IRB. The study participant MUST be consented with the EC/IRB-approved ICF before the participant is subjected to any study procedures. The approved ICF MUST be signed and dated by the study participant or legally acceptable representative and the investigator-designated research staff obtaining the consent. Any revisions to the ICF at any time during the trial will need to be submitted to the IRB for approval and submission to ACRIN PDRC.

14.0 CONFLICT OF INTEREST

Any investigator and/or research staff member who has a conflict of interest with this study (such as patent ownership, royalties, or financial gain greater than the minimum allowable by their institution) must fully disclose the nature of the conflict of interest in accordance with ACRIN Conflict of Interest policies and applicable federal, state, and local laws and regulations.

15.0 PUBLICATION POLICY

Neither complete nor any part of the results of the study obtained under this protocol, nor any information provided to the investigator for the purposes of performing the study, will be published or passed on to any third party without the consent of ACRIN, the Study Chair, and/or ACRIN Publication Committee. Any investigator involved in this study is obligated to provide ACRIN with complete test results and all clinical data obtained from the participants in this protocol. Investigators will follow the ACRIN Publication Policy (available online at www.acrin.org/PublicationsPolicy.aspx).

16.0 INSTITUTIONAL MONITORING AND AUDITS

The investigator will permit study-related auditing and inspections of all study-related documents by the EC/IRB, government regulatory agencies, and ACRIN. The investigator will ensure the capability for inspection of all participating sites' study-related facilities (e.g. imaging centers, satellite sites). The investigator will allocate adequate time for these activities, allow access to all study-related documents and facilities, and provide adequate space to conduct these visits.

16.1 Monitoring

Monitoring ensures data quality and the rights, safety, and well-being of the participants are protected. Monitoring also makes certain that the trial is in compliance with the currently approved protocol/amendments, with GCP and applicable regulatory requirements. It will provide the site an opportunity to verify that reported trial data are accurate, complete and verifiable from source documents. Institutional monitoring will be implemented at several different time points during the conduct of the study.

Monitoring instructions will be sent to the site prior to the implementation of monitoring to aid in preparation for the review. The instructions will specify regulatory documents and participant

case records scheduled to be monitored. Case report forms (CRFs) and source documents of selected study participants enrolled at each site will be reviewed. In addition, the initial regulatory documents and any revised regulatory documents will also be monitored.

16.2 Audits

All participating institutions that enroll participants will be audited. The timing of the initial on-site audit will depend upon several factors, including the rate of accrual (both study-wide and site-specific), the number of evaluable participants enrolled at an individual site, the status of the protocol and pending amendments, and monitoring status. Generally, audits will be conducted after the number of evaluable participants reaches 30% of targeted accrual, either study-wide and/or site-specific. Audits are typically scheduled to occur at least 3 months after an institution has been monitored, providing that monitoring did not identify issues that mandate immediate auditing. This schedule may be altered in the event of pending protocol amendments. Closure of the study to accrual will trigger auditing of all participating institutions not yet audited. Additionally, site-specific circumstances may prompt an audit at any time.

Subsequent audits will be scheduled per the outcome of the initial audit. Audits can be conducted more frequently at the discretion of the protocol team. The audits will be conducted per procedures established by the Cancer Imaging Program (CIP) of the NCI. Instructions for preparation for the audit visit will be sent to the site prior to the scheduled audit visit. These instructions will specify which participant case records will be reviewed during the audit. On-site records will be verified against the submitted form, and the findings will be recorded on specially-prepared audit reports. Major discrepancies will be forwarded to the appropriate oversight body within ACRIN. IRB procedures, approvals, and ICFs will also be reviewed at the time of the audit visit. The ACRIN Audit Manual is available online at www.acrin.org/pdrc.aspx.

To help sites prepare for monitoring and audits and to assure that the investigator and the research staff maintain records appropriately, ACRIN Headquarters will offer training to sites. This training will cover all aspects of data collection, including special instructions to obtain and file the various source documents needed to verify the accuracy of submitted data for this trial.

16.3 Source Documents

Source data are found in all information, original records of findings, observations, or other activities in a clinical trial necessary for the reconstruction and evaluation of the trial. Source data are contained in source documents. Source documents represent the first recording of any observations made or data generated about a study participant while he or she is enrolled in a clinical trial. Source documents for each study participant substantiate the data that are submitted to ACRIN. Source documents must verify the eligibility criteria and data submitted on all CRFs.

Research records for each case should contain copies of the source documents for the data collected and reported to ACRIN. If data are abstracted from medical charts that are not filed at the investigative sites (e.g. hospital charts), copies of these records should be filed in the research chart. Every attempt must be made to obtain all records/charts that were used to abstract any study data for this protocol. This will prevent any discrepancies and the inability to verify the document and the data reported.

16.4 Case Report Forms (CRFs)

CRFs, both web-based and paper forms, are the primary data collection instruments for the study. All data requested on the CRFs must be recorded, and any missing data must be explained. If a space is left blank because the procedure was not done or the question was not asked, “N/D” must be noted. If the item is not applicable to the individual case, “N/A” must be noted. All entries must be printed legibly in black ink on the paper CRFs. In the event of any entry errors, corrections must be made by drawing a **single straight line** through the incorrect entry, writing **the initials of the person making the correction, recording the date** when the correction is being made, and entering the correct data above the strike through. Do not use white out or an eraser. Please refer to ICH Good Clinical Practice Guidelines.

Data elements that are extracted from the medical record (source documents such as participant history or official clinical interpretations of images, pathology, or surgery results) and recorded on the CRFs will be reviewed against the appropriate component of the medical record. Data elements gathered from signed participant questionnaires must be available for review. Required study image interpretation data that are more detailed in information than the image and not typically documented in the standard radiology report may be documented on the CRF; the CRF is therefore acceptable source documentation **if signed by the Investigator**.

At the time of audit, the auditor will verify the occurrence of the imaging examination, the reader, and the date(s) of the exam(s) from the medical record(s). Any use of approved CRFs as source documentation require a signature and date on the CRF with a reference to the information source (participant questionnaire, CT, MR, etc.). Any use of CRFs as source documentation when the protocol has designated the source data will be medical record documentation will be considered a major protocol deficiency.

16.5 Institutional Review Board

Sites must obtain initial local IRB approval to participate in ACRIN trials. Prior to participant registration, a copy of the IRB approval letter for the protocol and the ICF must be sent to ACRIN, along with a copy of the IRB-approved, site-specific ICF. Investigator will provide a copy(s) of IRB approval letter(s) for any amendment(s), and copy(s) of annual renewal(s).

17.0 STATISTICAL CONSIDERATIONS

This section has been intentionally left blank.

This section has been intentionally left blank.

This section has been intentionally left blank.

18.0 TISSUE SPECIMEN COLLECTION FOR BIOMARKER ANALYSIS

Tumor tissue submission is strongly encouraged but not a requirement due to site-specific guidelines on tissue distribution. As feasible for each participant, the tumor tissue obtained from the diagnostic biopsy or surgery for diagnosis of GBM will be collected, analyzed, and stored until processing at an ACRIN-designated pathology laboratory for purposes of analysis for tumor hypoxic and vasculature markers and MGMT testing. No additional study-specific biopsy will be obtained. Sites will be provided with specific detailed collection instructions and shipment information at the initiation of the trial.

18.1 Tissue Analysis for Tumor Hypoxic Markers and MGMT Testing

As feasible for each participant, at least 1 block of tumor tissue but preferably no less than 3 blocks with accompanying representative H&E slides and pathology reports, will be obtained for the correlative studies of MGMT status and hypoxia/vascular markers. **Blocks are strongly preferred.** Block tissue areas should ideally be >1cm^2. For MGMT testing, the selected block should be composed of mostly viable tumor cells (i.e. >70%) but for the hypoxia/vascular markers, blocks containing viable tissue and areas of necrosis are preferred. No block will be completely depleted of tissue and blocks containing residual tissue will be returned to the sites.

For a limited-size biopsy specimen and sites not approved to send blocks, **a minimum of twenty (20)** 5-µm thick (section areas >1cm^2), unstained sections on chemoplus/plus slides will be acceptable and are requested.

A minimum of ten (10) slides for each analysis are needed for the two analyses to be performed:

- At least 10 slides composed of mostly viable tumor cells (i.e., >70%) for MGMT testing. If tissue area is <1cm^2, an appropriate number of additional slides are requested in order to obtain an amount of tissue similar to that afforded by ten (10) 5-µm thick sections at >1cm^2.

- Ten (10) additional slides are requested for immunohistochemical analyses; these sections should ideally contain viable tumor tissue and areas of necrosis.

Blocks or slides, representative H&E slides, and pathology reports should be sent to:

Dr. Catherine Nutt
Molecular Pathology Unit, Massachusetts General Hospital
Room 6014
Building 149, Thirteenth Street
Charlestown, MA 02129

18.1.1 Hypoxia and Vascular Markers

To investigate the possible relationships between hypoxic markers and FMISO uptake with progression-free survival and overall survival, tumor tissue obtained for diagnosis of glioblastoma will be analyzed for expression of hypoxic and vascular markers. No additional biopsy will be obtained. In particular, HIF1-alpha, GLUT1, CAIX, CD31, and alpha-SMA, will be assessed using immunohistochemical staining. HIF1-alpha, GLUT1, and CAIX will be used to evaluate degree of hypoxia, and CD31 and alpha-SMA will be used to look at tumor blood vessels and pericyte coverage.

Briefly, 5-μm sections will be cut from selected blocks. The sections will be deparaffinized in xylenes and rehydrated through graded alcohols. Endogenous peroxidase activity will be quenched with 0.5% H_2O_2 in methanol for 10 min during one of the rehydration steps. Antigen unmasking will be performed by heating slides immersed in sodium citrate or EDTA buffers, according to antigen retrieval protocols previously optimized for each antibody. The slides will then be allowed to cool in the buffer for 20 min. To block nonspecific immunobinding sites, slides will be incubated in 10% NHS in PBS/1% BSA for 30 min at room temperature; in addition, 5% milk is added to the blocking step for alpha-SMA.

Immunostaining will be performed by incubation with the appropriate primary antibodies (monoclonal or polyclonal) for the optimized time and dilution. Each round of staining includes a positive control tissue as well as the positive control tissue incubated without primary antibody as a negative control. This also controls for round-to-round variation in staining so that samples can be better compared across multiple rounds of staining performed over time. The slides will be washed with phosphate-buffered-saline (PBS) and then incubated for 30 min with biotin-conjugated secondary antibody in PBS/1%BSA; concentrations of secondary antibodies vary for each antibody according to previously optimized protocols. Antibody binding sites will be visualized by avidin-biotin horseradish peroxidase complex solution (Vectastain ABC kit, Vector Laboratories) and 3, 3'-diaminobenzidine (DAB). After rinsing in water, the slides will be counterstained with Gills hematoxylin (Sigma Chemicals, St. Louis, MO), dehydrated, and coverslipped.

Immunostains of the biopsy specimens will be scored according to antibody-dependent protocols. Briefly, HIF1-alpha will be scored separately for the percentage of tumor cells positive for cytoplasmic (non-activated) and nuclear (activated) staining; GLUT1 and CAIX will be scored for the percentage of tumor cells demonstrating positive membrane staining; CD31 and alpha-SMA staining will be scored by standard microvascular density

techniques in the areas of highest vessel density. Subsequent to detailed scoring of the tissues, refined scores of 0-3+ will be assigned based on appropriate antibody-dependent scales, as previously determined. The immunostain results will be interpreted by Dr. Nutt, who will be blinded to the imaging results and will assign each biopsy with a composite score for each marker.

18.1.2 MGMT Testing

MGMT analysis will be performed by extracting tumor genomic DNA for bisulfite conversion (EZ DNA Methylation-Gold Kit, Zymo Research, Orange, CA). *MGMT* methylation-specific PCR (MSP) will then be performed using PCR primer sets specific for methylated and unmethylated *MGMT* promoter sequence.[39] Presence of unique PCR amplification products generated by the "methylated MGMT" and "unmethylated MGMT" primers is assessed along with appropriate bisulfite-treated methylated and unmethylated control genomic DNA using agarose electrophoresis.

REFERENCES

1. Stupp R, Mason WP, van den Bent MJ, et al. Radiotherapy plus concomitant and adjuvant temozolomide for glioblastoma. *N Engl J Med*. 2005;352:987-996.
2. Dewhirst MW, Cao Y, Moeller B. Cycling hypoxia and free radicals regulate angiogenesis and radiotherapy response. *Nat Rev Cancer*. 2008;8:425-437.
3. Spence AM, Muzi M, Swanson KR, et al. Regional hypoxia in glioblastoma multiforme quantified with [^{18}F]fluoromisonidazole positron emission tomography before radiotherapy: Correlation with time to progression and survival. *Clin Cancer Res*. 2008;14:2623-2630.
4. Bruehlmeier M, Roelcke U, Schubiger PA, Ametamey SM. Assessment of hypoxia and perfusion in human brain tumors using PET with ^{18}F-fluoromisonidazole and 15O-H2O. *J Nucl Med*. 2004;45:1851-1859.
5. Tochon-Danguy HJ, Sachinidis JI, Chan F, et al. Imaging and quantitation of the hypoxic cell fraction of viable tumor in an animal model of intracerebral high grade glioma using [^{18}F]fluoromisonidazole (FMISO). *Nucl Med Biol*. 2002;29:191-197.
6. Valk PE, Mathis CA, Prados MD, et al. Hypoxia in human gliomas: demonstration by PET with fluorine-18-fluoromisonidazole. *J Nucl Med*. 1992;33:2133-2137.
7. Cher LM, Murone C, Lawrentschuk N, et al. Correlation of hypoxic cell fraction and angiogenesis with glucose metabolic rate in gliomas using ^{18}F-fluoromisonidazole, ^{18}F-FDG PET, and immunohistochemical studies. *J Nucl Med*. 2006;47:410-418.
8. Rasey JS, Nelson NJ, Chin L, et al. Characteristics of the binding of labeled fluoromisonidazole in cells in vitro. *Radiat Res*. 1990;122:301-308.
9. Gross MW, Karbach U, Groebe K, et al. Calibration of misonidazole labeling by simultaneous measurement of oxygen tension and labeling density in multicellular spheroids. *Int J Cancer*. 1995;61:567-573.
10. Koh WJ, Rasey JS, Evans ML, et al. Imaging of hypoxia in human tumors with [F-18]fluoromisonidazole. *Int J Radiat Oncol Biol Phys*. 1992;22:199-212.
11. Bernsen HJ, Rijken PF, Peters H, et al. Hypoxia in a human intracerebral glioma model. *J Neurosurg*. 2000;93:449-454.
12. Rasey JS, Koh WJ, Evans ML, et al. Quantifying regional hypoxia in human tumors with positron emission tomography of [18F]fluoromisonidazole: a pretherapy study of 37 patients. *Int J Radiat Oncol Biol Phys*. 1996;36:417-428.
13. Rasey JS, Grunbaum Z, Magee S, et al. Characterization of radiolabeled fluoromisonidazole as a probe for hypoxic cells. *Radiat Res*. 1987;111:292-304.
14. Troost EG, Laverman P, Kaanders JH, et al. Imaging hypoxia after oxygenation-modification: comparing [18F]FMISO autoradiography with pimonidazole immunohistochemistry in human xenograft tumors. *Radiother Oncol*. 2006 Aug;80(2):157-64.
15. Dubois L, Landuyt W, Haustermans K, et al. Evaluation of hypoxia in an experimental rat tumour model by [(18)F]fluoromisonidazole PET and immunohistochemistry. *Br J Cancer*. 2004 Nov 29;91(11):1947-54.

16. Sorensen, M., Horsman, M. R.,Cumming, P., Munk, O. L.,Keiding, S. Effect of intratumoral heterogeneity in oxygenation status on FMISO PET, autoradiography, and electrode Po2 measurements in murine tumors. *Int J Radiat Oncol Biol Phys*. 2005;62(3):854-61.

17. Gagel B, Piroth M, Pinkawa M, et al. pO polarography, contrast enhanced color duplex sonography (CDS), [^{18}F] fluoromisonidazole and [^{18}F] fluorodeoxyglucose positron emission tomography: validated methods for the evaluation of therapy-relevant tumor oxygenation or only bricks in the puzzle of tumor hypoxia? *BMC Cancer*. 2007;7:113.

18. Ceelen W, Smeets P, Backes W, et al. Noninvasive monitoring of radiotherapy-induced microvascular changes using dynamic contrast enhanced magnetic resonance imaging (DCE-MRI) in a colorectal tumor model**.** *Int J Radiat Oncol Biol Phys*. 2006;64(4):1188-1196. (ePub 2006Feb2.)

19. Rauscher A, Sedlacik J, Barth M, Haacke EM, Reichenbach JR. Noninvasive assessment of vascular architecture and function during modulated blood oxygenation using susceptibility weighted magnetic resonance imaging. *Magn Reson Med*. 2005 Jul;54(1):87-95.

20. Taylor NJ, Baddeley H, Goodchild KA, et al. BOLD MRI of human tumor oxygenation during carbogen breathing. *J Magn Reson Imaging*. 2001;14(2):156-163.

21. Plate KH, Breier G, Weich HA, Risau W. Vascular endothelial growth factor is a potential tumour angiogenesis factor in human gliomas in vivo. *Nature*. 1992;359(6398):845-848.

22. Jensen RL, Ragel BT, Whang K, Gillespie D. Inhibition of hypoxia inducible factor-1alpha (HIF-1alpha) decreases vascular endothelial growth factor (VEGF) secretion and tumor growth in malignant gliomas. *J Neurooncol*. 2006;78(3):233-247.

23. Forsythe JA, Jiang BH, Iyer NV, et al. Activation of vascular endothelial growth factor gene transcription by hypoxia-inducible factor 1. *Mol Cell Biol*. 1996;16(9): 4604-4613.

24. Folkman J. Angiogenesis: an organizing principle for drug discovery? *Nat Rev Drug Discov*. 2007;6(4): 273-286.

25. Vredenburgh JJ, Desjardins A, Jerndon JE 2nd, et al. Phase II trial of bevacizumab and irinotecan in recurrent malignant glioma. *Clin Cancer Res*. 2007;13(4):1253-1259.

26. Friedman HS, Prado MD, Wen PY, et al. Bevacizumab alone and in combination with irinotecan in recurrent glioblastoma. *J Clin Oncol*. 2009;27(28):4733-4740.

27. Batchelor TT, Sorensen AG, diTomaso E, et al. AZD2171, a pan-VEGF receptor tyrosine kinase inhibitor, normalizes tumor vasculature and alleviates edema in glioblastoma patients. *Cancer Cell*. 2007;11(1):83-95.

28. Helleday T, Bryant HE, Schultz N. Poly(ADP-ribose) polymerase (PARP-1) in homologous recombination and as a target for cancer therapy. *Cancer Res*. 2005; 4:1176-1178.

29. Masutani M, Nakagama H, Sugimura T. Poly(ADP-ribosyl)ation in relation to cancer and autoimmune disease. *Cell. Mol. Life Sci*. 62; 769-783, 2005.

30. Wielckens K, Garbrecht M, Kittler M, Hilz H. ADP-ribosylation of nuclear proteins in normal lymphocytes and in low-grade malignant non-Hodgkin lymphoma cells. *Eur J Biochem*. 1980;104(1):279-287.

31. Hirai K, Ueda K, Hayaishi O. Aberration of poly(adenosine diphosphate-ribose) metabolism in human colon adenomatous polyps and cancers. *Cancer Res*. 1983;43(7):3441-3446.

32. Tomoda T, Kurashige T, Moriki T, et al. Enhanced expression of poly(ADP-ribose) synthetase genre in malignant lymphoma. *Am J Hematol*. 1991;37(4):223-227.

33. Shiobara M, Miyazaki M, Ito H, et al. Enhanced polyadenosine diphosphate-ribosylation in cirrhotic liver and carcinoma tissues in patients with hepatocellular carcinoma. *J Gastroenterol Hepatol*. 2001;16(3):338-344.

34. Tentori L, Leonetti C, Scarsella M, et al. Brain distribution and efficacy as chemosensitizer of an oral formulation of PARP-1 inhibitor GPI 15427 in experimental models of CNS tumors. *Int J Oncol*. 2005;26(2) :415-422.

35. Donawho CK, Luo Y, Luo Y, et al. ABT-888, an orally active poly(ADP-ribose) polymerase inhibitor that ptentiates DNA-damaging agents in preclinical tumor models. *Clin Cancer Res*. 2007;13(9):2728-2737.

36. Tentori L, Portarena I, Torino F, et al. Poly(ADP-ribose) polymerase inhibitor increases growth inhibition and reduces G(2)/M cell accumulation induced by temozolomide in malignant glioma cells. *Glia*. 2002;40:44-54.

37. Curtin NJ, Wang LZ, Yiakouvaki A, et al. Novel poly(ADP-ribose) polymerase-1 inhibitor, AG14361, restores sensitivity to temozolomide in mismatch repair-deficient cells. *Clin Cancer Res*. 2004;10:881-889.

38. Macdonald DR, Cascino TL, Schold C, Cairncross JG. Response criteria for phase II studies of supratentorial malignant glioma. *J Clin Oncol*. 1990;8:1277-1280.

39. Esteller M, Hamilton SR, Burger PC, Baylin SB, Herman JG. Inactivation of the DNA repair gene O6-methylguanine-DNA methyltransferase by promoter hypermethylation is a common event in primary human neoplasia. *Cancer Res.* 1999;59: 793-797.

APPENDIX I

ACRIN 6684

MULTICENTER, PHASE II ASSESSMENT OF TUMOR HYPOXIA (LOW OXYGEN) IN GLIOBLASTOMA BRAIN TUMORS USING THE INVESTIGATIONAL ^{18}F-FLUOROMISONIDAZOLE (FMISO) AGENT WITH PET AND MRI SCANS

INFORMED CONSENT FORM TEMPLATE

[Note: The American College of Radiology Imaging Network (ACRIN) does not monitor compliance with the Health Insurance Portability and Accountability Act (HIPAA); that is the responsibility of local Institutional Review Boards (IRBs). Local IRBs may choose to combine the authorization elements in the informed consent. Information on ACRIN's HIPAA policy, as well as a template for HIPAA authorization, can be found at www.acrin.org.]

This is a clinical trial, a type of research study. Your study doctor will explain this clinical trial to you. Clinical trials include only participants who choose to take part. Please take your time with your decision to take part. You are encouraged to discuss all parts of this study with your family and friends and to ask your healthcare team as many questions as needed.

If you want more information about participating in clinical trials, ask your study doctor for the National Cancer Institute (NCI) booklet *Taking Part in Cancer Treatment Research Studies*. Also, you can learn more about clinical trials at http://cancertrials.nci.nih.gov or by calling the NCI's help line at 1-800-4-CANCER (1-800-422-6237 or TTY: 1-800-332-8615).

You are being asked to be in this study because you have been diagnosed with a form of brain cancer called *glioblastoma*. This clinical trial involves taking pictures of the brain with imaging technology. Two types of images will be taken at different times during your brain cancer treatment. Contrast-enhanced magnetic resonance imaging (MRI) is used routinely to create pictures of the brain. This study will also be using positron emission tomography (PET) scans with a new investigational imaging agent that the Food and Drug Administration (FDA) is studying. If you decide to volunteer for this study, you will be asked to sign and date this form.

WHY IS THIS STUDY BEING DONE?

The purpose of this study is to see if the investigational radioligand ^{18}F-fluoromisonidazole (commonly known as FMISO) used during PET scans can help doctors see if a tumor is getting enough oxygen. Radiation and chemotherapy do not appear to work as well on tumors with low oxygen levels.

This clinical trial will study brain images from PET scans using the investigational radioligand FMISO. A *radioligand* is a molecule that carries a small amount of radioactive substance in to the body, where the PET scanner can pick up the radiation being released to create a picture from within the body. When the study doctors take pictures of the brain using a PET scanner after the FMISO agent has been injected into the body, they hope to see how well the tumor is getting oxygen.

If a brain tumor is not getting enough oxygen (called *hypoxia*), then the treatments that can destroy it might not work as well. The more doctors know about how the amount of oxygen a tumor gets effects treatment, the more likely they are to give a patient the right treatment from the beginning. This study will add to doctors' knowledge of how tumors, hypoxia, and treatments are related.

FMISO is not approved yet by the FDA for routine use in people diagnosed with brain cancer. This trial's results will add to the evidence available on this agent as the FDA decides whether it should be used regularly to help people with brain cancer.

<u>About MRI Scans With Gadolinium Contrast</u>

An MRI uses powerful magnets and radio waves linked to a computer to create cross-sectional images of the body, in this case of the brain, that are remarkably clear and detailed. The MRI scans taken in this study will help the study doctors view your brain tumor. A contrast agent called gadolinium will be used during your MRI scans. A *contrast agent* is a liquid-like dye that goes into the body to help imaging machines create pictures of the body's organs and bones.

<u>About PET Scans</u>

PET scan is a nuclear medicine imaging technique that produces a 3-D image of how the body functions. In other words, PET scans take pictures of the cells and how they work in the body—in this case, in the brain and, with the FMISO, how much oxygen is getting into the brain tumor.

<u>About the Radioligand FMISO</u>

The FMISO agent is an investigational radioligand, a molecule containing radioactive substance. The low dose of radiation that it gives off from inside the body is picked up on PET scan images. The images show color where the FMISO emits radiation so study doctors can see whether oxygen is available to different areas of the tumor.

HOW MANY PEOPLE WILL TAKE PART IN THE STUDY?

About 50 people newly diagnosed with advanced brain cancer (glioblastoma) from 4 to 10 institutions will take part in this study.

HOW LONG WILL I BE IN THE STUDY?

You will be directly involved with this clinical trial's imaging procedures for the two (2) weeks after you are enrolled to participate, during the time before you start treatment. You may sign this consent form and then be unable to participate because of new information about your health; in these instances, you will be informed and the reason you were unable to participate will be collected. However, information about your cancer—including whether it gets worse—will be collected from your treating doctor(s) until the study ends, which may be up to 5 years. The FMISO PET and MRI scans in this study will be taken as you undergo different stages of treatment for your brain tumor.

This study is expected to end after all study participants have completed the imaging visits and all the information has been collected. This study may be stopped at any time by your study doctor, ACRIN, FDA, or NCI without your consent should:

- Your health or safety be at risk;
- You not follow study instructions;
- The study drug no longer be available;
- New information becomes available that might change your mind about participating in the trial;
- An administrative decision made by the study doctor, ACRIN, FDA, or NCI.

These actions do not require your consent, but you will be informed of any of these decisions if such a decision is made.

You can stop participating in this study at any time. However, if you want to stop participating in the study, we encourage you to talk to the study doctor and your treating doctor before making your final decision. Withdrawal will not interfere with your future care. There will be no penalty for deciding not to participate.

WHAT AM I BEING ASKED TO DO IN THE STUDY?

If you agree to take part in this study and are determined to be eligible by your study doctor, you will be asked to read and sign this consent form before you are enrolled to participate in this trial and before any study procedures are performed. When you are enrolled into the study, you will have the following tests and procedures. As part of this study, a sample of your tumor tissue obtained from a biopsy or surgery that resulted in the diagnosis of a brain tumor will be sent and stored until processed at ACRIN designated pathology laboratory for analysis as part of this study. You will not need to have any additional biopsies to participate in the study.

See the Study Chart at the end of this section for a visit-by-visit outline of what will be expected of you if you decide to participate in this trial.

Standard medical procedures that are part of regular cancer care and would probably be done even if you do not join the study:

- Chemotherapy (cancer fighting drug) called temozolomide and possibly an additional medication ;
- Radiation therapy;
- Standard-of-care imaging during your treatment and follow up;
- Short mental health examinations;
- Physical examination;
- Neurologic examination;
- Routine blood laboratory studies;
- Pregnancy test (if applicable).

* If you are taking an additional agent as part of another clinical trial, you will be agreeing to allow ACRIN and its affiliates involved in the ACRIN trial collect data from the other clinical trial on the type of treatment and its administration.

Standard medical procedures that are being done specifically because you are in this study (these may or may not be done if you were not in this study):

- Blood samples will be taken each scanning visit during the trial;
- One (1) or two (2) PET scans with FMISO—if you are one of the 15 participants to be asked to have the extra imaging, you will have a total of 2 PET scans with FMISO;
- Additional imaging related to the pre-treatment MRI scan (this will mean more time than usual in the MRI machine);
- Your kidney function will need to be tested within 28 days prior to each study MRI scan.

Before treatment. You will be asked to have one (1) FMISO PET scan and one (1) MRI scan with contrast within the two (2) weeks prior to starting treatment for your brain cancer.

A group of 15 participants will also be asked to have one (1) FMISO PET scan within one (1) week prior to starting treatment. Those 15 people will get two (2) FMISO PET scans prior to starting treatment.

Please circle your answer.

If I qualify, I choose to take part in the test-retest substudy that is being done for research and that will require a second FMISO PET scan prior to beginning treatment.<br><br>**Yes** **No** ____________<br>Your Initials

Intravenous (IV) catheters. Each visit when you have the FMISO PET scan, you will need to have two (2) IV catheters placed, one in a vein in your left arm, the other in a vein in your right arm. During the FMISO PET scan, blood samples will be taken from one of the IV catheters. The other IV catheter will be used to inject the radioligand FMISO for the PET scan or the gadolinium contrast for the MRI scan. Two (2) IV catheters will be important to ensure that the blood samples taken during the study do not contain any of the FMISO contrast agent.

Note that if you have the MRI scan on a separate day from the FMISO PET scan, then you will need only one (1) IV catheter that day. You and your study and/or treating doctors should discuss the risks and benefits associated with two-days of scanning, as you would be exposed to additional risk from one more IV catheter placement for each additional imaging day throughout the study.

Preparation for a PET scan. Before each FMISO PET scan, do not deliberately fast and drink lots of liquids. You will be given details of what to do to prepare for your PET scan prior to each imaging visit.

During the PET exam. Each day of your FMISO PET scan, you first will be given an injection of a small amount of the radioactive drug/tracer into a vein in your arm or hand. The amount of radiation is small, about the same as x-ray studies like CT. The radioactive tracer only stays in your body for a few hours. The FMISO will travel to your brain to aid in the images created by the PET scanner.

During the PET scan, small amounts of blood will be collected from the IV catheter in the arm that did not receive the FMISO agent.

The PET scanner is a large machine with a hole in the middle. It looks like a donut with a table in the middle. Almost two hours (110 minutes) after the injection of FMISO, you will be asked to go to the bathroom (urinate) and then lie on a partially enclosed scanning table, face up. The table will slide into the machine. You will be asked to remain still during the scan. You will hear buzzing or clicking sounds during the scan. You will need to lie still for about 20 minutes before coming off of the scanning table.

The size of the opening is 27 to 30 inches. How much space you feel you have around you will depend on your body size and the scanner type. If you feel any anxiety over being in enclosed spaces, let your study doctor know. A mild sedative may be used to help you feel more comfortable during the exam.

Time required. The entire FMISO PET scan procedure is expected to take no more than 3 hours from the time of the FMISO injection. MRI scans can take approximately 90 minutes. Once you have had your FMISO PET scan(s), you will receive radiation and chemotherapy (also known as chemoradiation) about a week after completing your first scan(s). The chemoradiation you will be getting is not a part of this study. It will be the usual treatment for your kind of brain cancer. You and your treating doctor will decide how much chemoradiation you will get.

Follow up. You will continue to see your treating doctor once the chemoradiation treatments have ended. You will see your treating doctor at regular intervals according to her/his recommendations and usual practice. Information gathered by your treating doctor as part of your normal follow-up visits will be given to your study doctor or research staff so they can find out more about your health. Any MRI or CT scans that are performed as routine care during the study period will be submitted along with your health status to ACRIN Headquarters in Philadelphia, PA. Your personal information will not be included on the images, though, to protect your privacy.

Your treating doctor will be asked to inform the study doctor or research staff about your health and your disease status until the end of the study (up to five [5] years after you completed your treatment and had your last FMISO PET scan). The study doctors will want to collect follow-up images (standard of care MRI and CT scans) during this time. Your follow-up care will be decided between you and your treating doctor.

STUDY CHART

**VISIT 1: Registration/Screening**	• Sign the informed consent form;<br>• Have a physical examination;<br>• Provide medical history and take short mental health examinations;<br>• Routine lab studies, including checking your kidney health, if necessary;<br>• Have a pregnancy test, if necessary.
**VISIT 2: Baseline—Pre-Treatment FMISO PET and Brain MRI With Contrast**<br><br>***(Within 2 Weeks Prior to the Beginning of Treatment)***	• Follow instructions in preparation for the PET and MRI scans;<br>• Have a pregnancy test, if necessary;<br>• Check your kidney health, if necessary;<br>• Have vital signs taken before the FMISO injection;<br>• Have 2 IV catheters placed, one in a vein in each arm;<br>• Have the FMISO radioligand injected;<br>• Have a PET scan;<br>• Small amounts of blood (about 18–30 mL total—about 1 ounce) will be taken during the PET scan;<br>• Have vital signs taken after the PET scan is over;<br>• Describe your steroid use/dose prior to MRI;<br>• Undergo MRI scan with gadolinium contrast agent, injected while in the scanner;<br>• The day after the PET scan, a researcher will call you to see how you are feeling.<br><br>**NOTE:** You and your doctor have the option of scheduling 2 days to complete the FMISO PET and MRI scans. These scans would need to be completed within the same 14-day period prior to start of treatment.
**VISIT 2A: FOR 15 PARTICIPANTS ONLY – Reproducibility Arm for PET**<br><br>***(1 to 7 Days After Completion of Visit 2 Scans, and Within 1 Week Prior to the Beginning of Treatment)***	• Follow instructions for preparation for the PET scan;<br>• Have a pregnancy test, if necessary;<br>• Have vital signs taken before the FMISO injection;<br>• Have 2 IV catheters placed, one in a vein in each arm;<br>• Have the FMISO radioligand injected;<br>• Have a PET scan;<br>• Small amounts of blood (about 18–30 mL total) will be taken during the PET scan;<br>• Have vital signs taken after the PET scan is over;<br>• The day after the PET scan, a researcher will call you to see how you are feeling.<br><br>**NOTE:** Visit 2A must be completed 1 to 7 days after the completion of *both* Visit 2 scans (FMISO PET and MRI) and before the start of treatment.
**FOLLOW UP: Every Three Months Until the End of Study (5 Years at Most)**	• Every three months after the imaging for this study is completed, a researcher will contact your treating physician to collect copies of any MRIs or CT scans taken during routine follow up.<br>The researcher will also ask about your condition, including whether you have received any new treatments, are taking steroids for your disease, and whether your tumor has grown or shrunk.

WHAT ARE THE POSSIBLE RISKS OR DISCOMFORTS OF THE STUDY?

While on the study, you may be at risk for side effects. You should discuss the possible side effects listed below with the study doctor, research staff, and/or your treating doctor. There also may be other side effects that we cannot predict. Other drugs may be given to make side effects less serious and uncomfortable. Many side effects go away shortly after the imaging scan is stopped and IV catheters are removed, but in some cases side effects can be serious, long lasting, or permanent.

If you receive Ativan or another sedative, there is a small risk of a reaction to the sedative medication. We will not administer this drug if you have had a bad reaction to similar medications in the past or if your medical history suggests that you are at risk for a reaction. If you receive a sedative, you will not be allowed to drive yourself home.

Risks Associated With an IV Injection:

- Bruising, pain, or infection at the injection site;
- Leaking of IV fluid into tissues near the injection;
- Allergic reaction, which could be serious or life threatening.

Risks Associated With MRI:

- Anxiety/stress;
- Claustrophobia;
- Discomfort.

Because of the powerful magnetic force of the MRI scanner, you may not be able to participate in the study if you have:

- Metallic or other surgical implants (for example: pacemaker, heart valves, aneurysm clips, metal plates or pins and some orthopedic prostheses);
- Metal pieces in your eye(s) or other body part(s); or
- Difficulty lying still or inability to lie on your stomach.

Notify your doctor if any of the above relate to you. Also, carefully read the information you should receive at the MRI facility about other risks.

While there are no significant risks from MRI, you may be uncomfortable due to the loud noise and/or feelings of claustrophobia during the MRI. If you experience a sensation of claustrophobia while in the magnet, the MRI will be immediately stopped. You will be excluded from the study if you are pregnant or have a pacemaker or other electromagnetic device, or have a vascular clip in your head. No serious biologic effects have been reported from the magnetic fields used in clinical MRI.

Risks Associated With Gadolinium:

Approximately two percent (2%) of participants experience some side effects with the use of gadolinium; however, they are mostly mild *(nausea, headache, hives, temporary low blood pressure)*. Serious side effects are very rare and are discussed below. Recent information has suggested that the use of gadolinium may contribute to kidney disease in patients with poor kidney function. Therefore, we

will monitor your kidney function closely while you participate in this study. If there is any change in your kidney function, we may have to remove you from the study.

Less likely
- Headaches;
- Nausea.

Less likely, but serious
- Allergic reaction.

Very rare, but serious
- Nephrogenic systemic fibrosis (NSF)/Nephrogenic Fibrosing Dermopathy (NFD). NSF is a condition associated with the gadolinium contrast agent when there is severe kidney disease. Symptoms include tightening or scarring of the skin and organ failure. In some cases, it can be deadly. NSF has not been seen in patients with normal working kidneys or mild problems in kidney function. Prior to study entry, we will determine if your kidneys are working properly in order to help make sure the gadolinium contrast agent is safe for you.

Risks Associated With PET Scans:

- Discomfort from lying still on your back for a total of about 20 minutes for each scan;
- Claustrophobia (feeling anxious and 'closed in').

Risks Associated With [^{18}F]FMISO PET/CT:

There are a few possible risks of [^{18}F]FMISO PET and PET/CT scans. The most common ones are not considered serious. Any serious risks of [^{18}F]FMISO PET or PET/CT scans are considered very unlikely. All of the known risks are described below.

Radiation Risk

<<Each site may need to modify this section to quote the correct PET and CT dosimetry for its own PET/CT and PET-only scanners in accordance with its own institutional policies and procedures. The following language and dosing range is an example only.>>

FOR EXAMPLE:

There are some risks from the PET scans used to monitor your tumor status and your response to treatment. These scans will expose you to radiation. If you live in the US, you receive about 300 millirem of radiation each year. It comes from space and the earth around you. This is called "background radiation." A "millirem" (mrem) is a unit used to measure doses of radiation. The radiation dose to your whole body from each of your PET scans will range from *780* to *980* mrem for participants who have two total study-related FMISO PET scans (15 participants who have two FMISO baseline scans). For participants who undergo one total study-related FMISO PET scans, the likely doses will range from *390* to *490* mrem. This dose can vary from person to person. Each dose of FMISO will expose you to about one-half to three-quarters of the amount of radiation in a routine CT scan of a large body area such as your abdomen.

At a dose of 1500 mrem, your risk of harm might be as high as 1 in 1000. Most likely, the effects of this radiation exposure will be years away. If you have more procedures that expose you to radiation, your risk will go up. Risks of harm may include getting a new cancer or changes in your genes. You may

need to have other x-rays or scans for your care. Your doctors will explain the risks of the other x-rays or scans.

As with any experimental procedure, there may be unanticipated side effects. But if you notice anything differently, please feel free to contact the investigators (contact number given below).

Reproductive Risk

If you are pregnant or nursing or plan to become pregnant leading up to the imaging component of the study, you cannot take part in this research study. We do not know the effects of the FMISO agent on the fetus, breastfeeding baby, or mother-to-be, and this study may cause harm. Because the PET and MRI scans and the investigational radioligand FMISO in this study can affect an unborn or nursing baby, you should not become pregnant or breastfeed, or father a baby, while undergoing imaging with FMISO for this study.

You and your study doctor should discuss taking precautions. If you or your partner does become pregnant, you will need to tell your study doctor immediately. If you are unsure of your pregnancy status on the day of your imaging scans for the trial, you will need to tell your study doctor and have a pregnancy test before any of the day's study procedures.

Ask about counseling and more information about preventing pregnancy.

WHAT ARE THE POSSIBLE BENEFITS OF TAKING PART IN THE STUDY?

You will not receive any direct benefit from participation in this research study. We hope the information learned from this study will benefit other patients with brain cancer in the future. Doctors may be able to use the information from this study to help them determine the best treatment approaches for people with certain types of brain cancer or help treatments work better in brain cancers that are difficult to treat.

WHAT OTHER CHOICES DO I HAVE IF I DO NOT WANT TO PARTICIPATE?

You may choose to not participate in this study. You could have PET and MRI scans done without participating in this study. If you decide not to participate, there will be no penalty or loss of benefits to which you are otherwise entitled. Please talk with your treating doctor about this and other options.

WHAT ABOUT PRIVACY?

Every attempt will be made by the investigators to keep all the information collected in this study strictly private, including your personal information. We cannot guarantee absolute privacy. Records of your participation in this study, your progress, and images taken while on the study (such as the FMISO PET and MRI scans) will be kept secure at this institution and in a computer file at ACRIN headquarters in Philadelphia, PA. All data sent to ACRIN over the Internet will be coded so that other people cannot read it. Your personal information may be disclosed if required by law.

You further understand that authorized representatives of ACRIN, the Center for Statistical Sciences at Brown University, the FDA, the NCI, Cardinal Health (supplier of FMISO), the IRB of *<<Institution>>*, and other groups or organizations that have a role in this study will have access to and may copy both your medical and research records due to your participation in this study. This access is necessary to ensure the accuracy of the findings and your safety and welfare. If any publication or presentations result from this study, you will not be identified by name. Results will be reported in a summarized manner that you cannot be identified.

Your research records and images will be kept permanently on file at ACRIN and may be used for future research. All personal identifiers are removed and replaced with a unique identifying number. The information that may be done with the information will not specifically help you. But, it might help people in the future who have cancer and other diseases.

WILL I HAVE TO PAY FOR ANYTHING?

Taking part in this study will not lead to added costs to you or your insurance company.

You or your insurance company will not be charged for the following parts of this research study:

- Blood samples collected at each scanning visits;
- One (1) or two (2) PET scans with FMISO.

You or your insurance company will be charged for any other portion of your care that is considered standard of care for your condition. You may be responsible for any co-payments and deductibles that are standard for your insurance coverage.

WILL I BE PAID FOR BEING IN THE STUDY?

The National Cancer Institute (NCI) allows reasonable reimbursement for travel expenses and time away from work associated with trial participation. If you are eligible to enroll in the study, you will receive a total of *$<<institution to provide appropriate amount—sites can determine a per-visit rate based upon appropriateness for their institution >>* upon completion of the study as compensation for time and travel associated with your participation in this research study. You will have one (1) or two (2) FMISO PET scans for this trial; the number of unique visits depends on your standard-of-care treatment. If, for whatever reason, you do not complete all the research-related imaging scans, you will be given *$<<institution to provide appropriate amount—sites can determine a per visit rate based upon appropriateness for their institution>>* per each research-related scan completed.

WHAT HAPPENS IF I AM INJURED BECAUSE I TOOK PART IN THE STUDY?

It is important that you tell your study doctor, *<<insert name>>*, if you feel you have been injured because of taking part in this study. You can tell the study doctor in person or call him/her at *<<insert telephone number>>*.

In the case of medical emergency, injury, or illness during this study, emergency medical treatment is available but will be provided at the usual charge. You and/or your insurance will be responsible for the cost of the medical care of that illness or injury. The study will not pay for medical treatment.

WHAT ARE MY RIGHTS IF I TAKE PART IN THIS STUDY?

Taking part in this study is voluntary. You may choose not to take part in the study. If you decide to take part in the study, you are free to leave the study at any time. No matter what decision you make, there will be no penalty and you will not lose any of your regular benefits. Leaving the study will not affect your medical care. You can still be treated at our institution, and your decision will not interfere with your future care.

During the study, we may find out more information that could be important to you. A Data Safety and Monitoring Board, an independent group of experts, may be reviewing the data from this research throughout the study. This includes information that might cause you to change your mind about being in the study. If information becomes available from this or other studies that may affect your health, welfare, or willingness to stay in this study, we will tell you about it as soon as possible.

WHO CAN ANSWER MY QUESTIONS ABOUT THE STUDY?
(This section must be completed)
You can talk with your study doctor(s) about any questions or concerns you have about this study. Contact your study doctor *<<insert name>>* at *<<insert telephone number>>*.

This document explains your rights as a study participant. It you have any questions at any time regarding your participation in this research study or you have any questions regarding your rights as a research participant, do not hesitate to speak with your study doctor or anyone listed below.

For additional information about your health or medical emergency, you may contact: *Usually the name of the local hospital information is provided and with instructions to study participants to inform the ER doctor of their participation in a clinical trial.*

________________________	____________________
Name	Telephone Number

For information about your rights as a research subject, you may contact *<<Institution Name>>* Institutional Review Board (a group of people who review the research to protect your rights):
(Provide the name of local IRB contact person)

________________________	____________________
Name	Telephone Number

WHERE CAN I GET MORE INFORMATION?

You may call the NCI’s Cancer Information Service at
1–800–4–CANCER (1–800–422–6237) or **TTY: 1–800–332–8615**

You may also visit the NCI's Web sites for comprehensive clinical trials information, http://cancertrials.nci.nih.gov. For cancer information from the NCI, visit http://cancernet.nci.nih.gov. ACRIN's web site is www.acrin.org.

ACKNOWLEDGEMENT

When you sign this document, you are agreeing to take part in this study. This means you have read all the above information, asked questions regarding your participation, and received answers that you understand to all your questions. You have also had the opportunity to take this consent form home for review or discussion if you want to.

You willingly give your consent to participate in this study. A copy of this signed informed consent form will be given to you. You may also request a copy of the protocol (full study plan).

________________________________ ________________________________

Printed Name of Study Participant/ Legal Representative | Signature | Date

<Insert other signature and date lines as appropriate per local IRB policies and procedures>

APPENDIX II

ACRIN 6684

SUPPLEMENTAL MATERIALS AVAILABLE ONLINE

Supplemental materials that support the conduct of the trial are available on the ACRIN Web site at the ACRIN 6684 Protocol Web page (www.acrin.org/6684_protocol.aspx). Types of materials posted include:

- Application and protocol activation documents (General Qualifying and Protocol Specific Applications, Form FDA 1572, ACRIN Statement of Investigators, protocol activation checklist, etc.);
- Data forms;
- Imaging materials (Image Transmittal Worksheet, imaging parameter charts, and scanning and image qualification instructions), see www.acrin.org/6684_imagingmaterials.aspx;
- Recruitment and education materials;
- Regulatory resources;
- Participating site list.

For more information related to the trial, contact the ACRIN 6684 Contact Personnel link on the above-mentioned Web page for a list of protocol team members at ACRIN Headquarters and their roles.

APPENDIX III

ACRIN 6684

WELL COUNTER CROSS-CALIBRATION PROCEDURE

NOTE: As a pre-qualification measure, sites will need to demonstrate appropriate well counter cross calibration.

1. The time of all measurements should be documented in reference to a single clock. If possible, the clocks on the PET scanner, well counter, and dose calibrator (if it has a clock) should be synchronized to this reference clock.
2. Fill a uniform cylindrical phantom (18-22 cm diameter) with water and eliminate as many air bubbles as possible.
3. Fill a syringe with a sufficient activity of ^{18}F to achieve a concentration in the phantom of approximately 0.2 uCi/mL. Make sure the syringe is properly placed inside the dose calibrator. Assay the syringe activity in the dose calibrator and record the assay result in mCi and the assay time.
4. Withdraw approximately 60 cc of water from the phantom using a large syringe and an 18-gauge needle.
5. Add the ^{18}F into the phantom and thoroughly rinse the syringe contents into the phantom.. Assay the residual activity in the syringe in the dose calibrator and record the assay result and assay time, seal the phantom and repeatedly invert to mix the solutions. Enter the measured full syringe activity (mCi), measured full syringe activity assay time, residual syringe activity (mCi), residual syringe activity assay time, and volume of the completely filled phantom (mL) in the EXCEL spreadsheet.
6. Add water to the phantom to fill it completely. Re-seal and thoroughly mix the contents of the phantom, to make sure that the ^{18}F is uniformly distributed.
7. Using a calibrated balance (with at least 1 mg precision), weigh each of the three (labeled 1,2,3) gamma tubes with their respective caps, and enter the weight of each gamma tube (including cap) separately in the EXCEL spreadsheet.
8. Withdraw three 1.0 mL samples from the phantom with a syringe or pipette and place into the weighed gamma counting tubes from Step 7. Replace the cap on each gamma counting tube...
9. Re-seal the phantom and position in the center of the gantry using the phantom holder supplied with the scanner.
10. Perform a 20 minute static scan using identical acquisition and reconstruction parameters used for FMISO patient acquisitions.
11. Using the same calibrated balance as in step 7, weigh each of the gamma counting tubes containing the samples and enter the results in the EXCEL spreadsheet.
12. With a pipette or syringe, place 1.0 mL of tap water into a gamma counting tube labeled “BKG” for measuring background radiation.

13. Count each of the samples and the BKG tube in the well counter for 60 seconds using a counting window set for 511 keV photons, or close equivalent. Enter the counting results and start times of the counting of each sample into the EXCEL spreadsheet

14. The formulas in the spreadsheet will combine the weight of the samples and the number of counts per minute and display the well counter rate concentration, $R_{(wc)}$ (cpm/mL), decay corrected back to the start time of the PET scan. The cpm/mL is calculated by dividing by the net volume of the sample in the gamma counting tube, assuming a density of 1 g/mL.

15. Upon completion of the image reconstruction measure the mean SUV (based on weight) by centering a large circular ROI with a diameter that is at least 3 cm smaller than the diameter of the uniform cylindrical phantom, on one plane and then copying that ROI, to all planes (EXCEPT THE FIRST AND LAST PLANE). Average the mean SUV's for all the ROI's throughout the phantom as measured by the PET scanner.

16. Using the EXCEL spreadsheet provided, enter the data from the procedure above to compute the cross-calibration factor.

17. The calibration factor (CF) between the well counter (wc) and the Dose Calibrator (dc), and the calibration factor between the PET scanner and the DOSE Calibrator is calculated as follows:

$CF_{wc} = R_{(wc)}/R_{(wc)}$
$CF_{PET} = R_{(dc)}/PET$

Note: The units of CF are uCi/CPM, which is used by multiplying the blood sample CPM by CF_{wc} to convert the units to uCi/mL. The units of CF_{PET} are (uCi/mL)/SUV. The ACRIN Core PET Lab will complete the calculations when processing FMISO image and blood data.

Appendix IV

ACRIN 6684

BLOOD SAMPLING PROCEDURES

NOTE: As a pre-qualification measure, sites will need to demonstrate appropriate well counter cross calibration. Use of dose calibrators for this procedure is strictly prohibited as the dose calibrator is not sensitive enough for the following measurements.

1. Find a single global clock that can be used to record each count time. Synchronize all clocks to the PET scanner time prior to performing the procedures, especially the well-counter clock and the clock used to measure blood draw times.
2. The well counter being used should have a CF calibration within one week prior to the study. The value and the date of calibration must be entered on the appropriate data submittal form.
3. Three venous blood samples are obtained at intervals of five minutes (± two minutes) at 5, 10, and 15 minutes after the start of emission scanning. The exact time of the blood draws must be recorded.
4. The blood samples must be drawn from the IV placed in the opposite arm, which was not used for the FMISO injection.
5. To avoid dilution, 3 – 5 mL must be drawn for "waste" prior to each of the blood samples drawn.
6. To ensure adequate volume to pipette into the counting tubes, 3 – 5 mL must be drawn for each blood sample.
7. Prior to adding the blood sample to the gamma tube, the tube must be weighed. The weight must be recorded on the appropriate form.
8. After weighing the empty tube, approximately 0.5 mL will be pipetted into the gamma tube.
9. The weight of the filled tube will then be measured. Results must be recorded on the appropriate form.
10. Background counts must be measured in the well counter prior to counting each of the blood samples. The background count must be performed with nothing in the well counter for an interval of 2 minutes. Results must be recorded on the appropriate data submittal form.
11. Following the background measurement, the gamma tube containing the blood sample will be counted for 2 minutes. Results must be recorded on the appropriate form.
12. Steps 4 through 10 will be repeated for each of the three blood draw time points.

Appendix V

ACRIN 6684

MACDONALD RADIOGRAPHIC RESPONSE CRITERIA

Response	***Enhancing Tumor Area***	***Neurological Status***	***Steroids***
Complete Response	≥ 95% decrease	Improved or Stable	Off
Partial Response	50% – 94% decrease	Improved or Stable	Stable or Decreased Dose
Progressive Disease	≥ 25% increase	Worsened	Stable or Increased Dose
Stable Disease	All other situations	All other situations	All other situations

Source: Macdonald et al. Response criteria for phase II studies of supratentorial malignant glioma. *J Clin Oncol*. 1990; 8:1277-1280.

Estimation of Radiation Doses Due To PET Radiopharmaceuticals and to Attenuation Correction of Pet Images

There are multiple reasons that contribute to the various radiation doses reported with PET and PET/CT scanning. They can be attributed to:

1. Source of Attenuation Correction: The radiation dose due to attenuation correction varies widely based on the attenuation correction method used. This can be a low dose cesium or germanium source, a low dose or higher dose non-diagnostic CT scanner, a diagnostic CT scanner. The method of attenuation correction can be a major source of radiation dose variation. The CT scanner characteristics and recommended settings vary considerably between manufacturers and different centers use different scanning settings to meet the needs for their studies.

2. The PET tracer being evaluated: Different PET tracers have varying biodistribution as well as varying levels of uptake in areas of interest – such as a tumor. The tracer uptake can vary not only across histologies (different tumor types) but also in different grades of the same histology. Most of the studies that the NCI is performing are to define these attributes for PET tracers of interest in the relevant histologies, so different studies may require different doses of the same radiopharmaceutical in order to provide evaluable quantitative or qualitative output.

3. The timing of the scan: Given that these PET tracers have fairly short half lives, the dose of the PET tracer may vary depending on when the scan is performed relative to the injection to deliver information of biological relevance. The longer the time between injection and scanning, the larger the dose that needs to be administered.

4. The area of the body being evaluated: Depending on the biodistribution, metabolic and the excretory pathways for a PET tracer, different amounts of the PET tracer may need to be injected in order to obtain an image with sufficient signal for accurate image interpretation in different body areas. In addition, the amount of attenuation changes based on the body part being imaged – and the dose is varied accordingly – for example, a brain PET can be performed with a smaller dose than a body PET T due to less attenuation and a more limited imaging field.

5. The type of PET imaging being performed: Some studies require dynamic scans to determine tracer kinetics, while other measures only static tracer uptake across a larger area of the body. Different radiopharmaceutical doses may be needed for these different types of studies depending upon tracer kinetics, area of the body imaged, and quantitative imaging requirements

6. Institution radiation safety policies: Different radiation-safety committees and IRBs have different approaches to estimating and communicating total radiation dose. A given institution may have standardized values that they use for all PET scans or may demand protocol and instrument specific dose evaluation. They may then communicate the maximum dose possible or the likely dose or the range. In all cases each institution’s radiation safety committee and IRB have the ultimate responsibility to communicate the exposure in the informed consent to their patients.

Further details are provided below.

The radiation dose from a PET scan has two components. The first is the dose from the radioactive material used in the study. The second is the dose from attenuation correction, which is almost always performed, both in routine clinical practice and in research studies. Table 1 shows the reported effective dose (ED) from the radioactive isotope for the various radiopharmaceuticals that are being studied under IND by the Cancer Imaging Program of the Division of Cancer Treatment and Diagnosis at the National Cancer Institute, along with FDG for comparison. Since these are empirically determined values involving model fitting and calculations, other estimates may not be identical to these, but will be similar. The exposure from a typical 10 mCi dose is also given, but note that the dose for these radiopharmaceuticals can vary. FDG for example, is labeled for 8-12 mCi, but some clinicians give substantially higher doses.

Table 1. Radiation dose for F-18 PET radiopharmaceuticals (Effective Dose)

Emission Doses	ED* (mSv/MBq)	ED (mrem/mCi)	Dose (mSv) for 370 MBq (10 mCi)
**F-18 FDG**[2]	0.016	60.8	5.9
**F-18 Fluoride**[3]	0.024	88.8	8.9
**F-18 FMISO**[4]	0.015	55.5	5.6
**F-18 FLT**[5]	0.026	96.2	9.6
**F-18 FES**[6]	0.023	83.4	8.5

*The ED was calculated from the individual organ doses (from published data), multiplying each by their weighting factor (as per ICRP60 and ICRP80) and summing the results to get the ED.

Attenuation correction is usually performed for research scans and can be performed three ways:

- Rotating rods of Ge-68 or point sources of Cs-137
- Low dose, non-diagnostic CT
- Diagnostic CT

The CT methods can have a range of radiation doses depending on scanner type and setting. Furthermore, the overall scan can be performed for three different imaging regions, which affects the effective dose from the transmission scan, depending upon the extent of the body that is imaged:

- Torso Survey (oropharynx to mid-thigh)
- Single Axial Field of View
- Head Only

The non-diagnostic CT provides both attenuation correction and anatomic correlation, so that the lesions identified in the PET scan can be localized anatomically with greater resolution than provided by a PET scan with gamma-ray source attenuation. In practice, the non-diagnostic CT has mostly replaced the use

[2] Hays MT, J Nucl Med, 43:210, 2002
[3] ICRP publication 53; calculated by ICRP and published in ICRP publication 80 on page 114)
[4] Graham MM et al, J Nucl Med 38:1361, 1997
[5] Vesselle H et al, J Nucl Med 44:1482, 2003
[6] Mankoff DA et al, J Nucl Med 42:679, 2001; for 56 kg female

of the gamma ray source attenuation because of the decreased scanning time and the improved anatomic correlation.

The radiation dose from attenuation by rotating rod with Ge-68 is low compared to that from the radiopharmaceutical or the low dose CT and has been reported[7] as 0.02, 0.08 and 0.20 mSv per exam for brain, single slice, and whole body. The dose from Cs-137 is much less, with an ED of 0.011 mSv reported[8] for a whole body scan.

Additionally, the CT scans, regardless of classification as diagnostic or non-diagnostic, are not necessarily identical at different institution or with different scanners even at the same institution. These four factors – the specific radiopharmaceutical, type of attenuation correction, institution instrumentation/practices and anatomic coverage – lead to a large range of effective doses being reported for a given PET radiopharmaceutical scan.

With the assistance of David Mankoff, MD, PhD, University of Washington, Seattle, a survey was done of the current attenuation correction practices and effective radiation doses for five major cancer centers experienced in PET/CT. The sites provided the CT settings they typically use for low dose scans and in two cases for diagnostic clinical scans. Radiation dose was calculated using ImPACT[9]. This program is a tool for calculating patient organ and effective doses from CT scanner examinations. It makes use of the National Radiological Protection Board (NRPB) Monte Carlo dose data sets produced in report SR250 from the UK Health Protection Agency. SR250 provides normalized organ dose data for irradiation of a mathematical phantom by a variety of CT scanners. A summary of the survey results is shown in Table 2 and the parameters used in the calculations are given in the appendix. The radiation dose for the transmission scans with Ge-68 and Cs-137 are included for comparison.

These survey results will only really apply to the more advanced research-orientated centers, but those are also the ones most likely to be participating in these early phase trials, so we expect these data to be representative of good practices at centers likely to perform phase 2 trials with investigational imaging agents.

[7] Wu T et al, Eur J Nucl Med Mol Imaging 31:38-43 (2004)
[8] Schaefer A et al, Nuklearmedizen 39: 204-208 (2000) [German]
[9] Medical Devices Agency. ImPACT CT patient dosimetry calculator, version (0.99X). London, England: ImPACT, 2006.

Table 2. Radiation doses calculated with ImPACT for multiple sites and procedures

		Torso survey (mSv)	Single Chest Field (mSv)	Head Only (mSv)
**Research Scans (non-diagnostic)**				
**Site 1**		6.1	1.0	0.1
**Site 2**	GE Lightspeed	4.9	1.6	0.4
**Site 3**	Siemens Sensation 16	8.0	2.4	1.0
	GE Lightspeed	8.7	2.2	1.0
**Site 4**	Phillips Mx8000 IDT	5.2	1.6	
	Phillips Big Bore	3.6	1.0	
**Site 5**		8.6		
**Median**		6.1	1.6	0.7
**Mean**		6.4	1.6	0.6
**Range**		3.6-8.6	1.0 - 2.4	0.1 - 1.0
**Transmission Scans**				
**Ge-68**		0.2	0.08	0.02
**Cs-137**		0.01	NA	NA
**Clinical Scans (diagnostic)**				
**Site 1**		13	3.7	0.2
**Site 2**		13	4.1	1.1

From these data, the following observations can be made:

1. The largest radiation dose is from the diagnostic scans, approximated twice that for non-diagnostic torso scans, and 3-4 times that for single axial scan at the same institution. These values are similar to those reported in a European study[10] of 14 to 18 mSv ED for high quality diagnostic scans.
2. Torso scanning, whether for diagnosis or for attenuation, exposes the patient to much more radiation than either single slice or head scans.
3. The ED for a torso scan using the low-dose non-diagnostic CT is approximately equivalent to the ED for the radiopharmaceutical at 10 mCi dose.
4. Head scans expose the patient to the smallest dose.
5. For a given machine, increasing the kVp or the mAs increases the dose.

When the dose for the attenuation scans is combined with the dose for the radiopharmaceuticals, the range of exposure for a specific radiopharmaceutical can be wide. As a result, there can be protocol to protocol differences as well as site to site differences for the same protocol.

Another important factor is that different radiation-safety committees and IRBs may well have different approaches to estimating and communicating total radiation dose. While the ImPACT program from the

[10] Brix G, et al, J Nucl Med, 46:608-613 (2005)

Medicines Device Agency in the UK is one of the more commonly used methods of estimating CT dose, it is not the only one. A given institution may have standardized values that they use for all PET scans or may utilize protocol and instrument specific dose evaluation. They may then communicate the maximum dose possible or the likely dose. In all cases each institution's radiation safety committee and IRB have the ultimate responsibility to communicate the exposure in the informed consent to their patients.

As a result, in a specific protocol, we can include our best estimates in the trial protocol and also include them in a draft informed consent form, but institutions can, and should, modify these in accordance with their local policies and practices.
